# Trajectory Control for Vibrating Screen with Magnetorheological Dampers

**DOI:** 10.3390/s22114225

**Published:** 2022-06-01

**Authors:** Szymon Ogonowski, Piotr Krauze

**Affiliations:** Department of Measurements and Control Systems, Silesian University of Technology, Akademicka 16, 44-100 Gliwice, Poland; piotr.krauze@polsl.pl

**Keywords:** vibrating screen, vibration trajectory control, magnetorheological damper, semiactive suspension, dynamic model, Bouc–Wen model

## Abstract

The article presents a method of vibrating screen trajectory control based on MR (magnetorheological) dampers applied in a screen suspension. A mathematical description of the dynamic screen model was derived, and parameters of this model were estimated based on experimental data from a semi-industrial vibrating screen. The investigated screen included a single mechanical exciter with unbalanced masses, generating a circular vibration trajectory and operating with over-resonant frequency close to 19 Hz. It was experimentally tested in several phases of operation: start-up, nominal operation at a target vibration frequency and shutdown. The implemented screen model was further extended and included several MR dampers oriented horizontally and vertically in the form of Bouc–Wen models. The Bouc–Wen model was identified based on experiments carried out for an MR damper subjected to harmonic excitations generated by the MTS (material testing system). Dominant frequencies of excitation varied by up to 20 Hz during experiments. The main novelty of the reported solution is that according to the proposed control algorithm, the desired forces generated by MR dampers emulate an additional virtual mechanical exciter of the vibrating screen. In turn, it interacts with the available exciter, resulting in conversion of the trajectory from circular to linear, which was validated in the presented study. For the purpose of simulation accuracy, the desired control force was additionally limited within the simulator by MR damper dissipative domain, which maps the constraints of a semi-active damper. The presented approach allows one to obtain a close to linear trajectory with only one exciter and with semi-active control of suspension stiffness. The results were successfully repeated with different configurations of desired trajectory, indicating that the effectiveness of the desired linear trajectory generation depends on its orientation. The reported findings may lead to the design of new vibrating screen constructions, taking advantage of the semi-active control of a suspension in the attenuation of disturbance resulting from varying processed material parameters.

## 1. Introduction

Screening and sieving are some of the oldest and extensively used nowadays physical size separation methods for bulk materials [1,2]. The use of such methods is widely present in laboratories for the purpose of particle size distribution analysis, and in the wide range of industries, such as mining, aggregate production, recycling and mineral processing, pharmaceuticals, cosmetics and food, as a unit operation for large scale separation [3,4]. One of the most common devices being applied for the above processes is the vibrating screen. Market analyses indicate that whole vibrating screen market was worth 2.1 billion USD in 2019 and is projected to reach 2.7 billion USD by 2025. The compound annual growth rate (CARG) is projected to go from 3% to 7% during the period of 2019–2025, depending on the source [5,6]. Even the recent COVID-19 pandemic did not manage to dramatically change the predicted growth trend [5]. In the industry, two main classes of screens are used: resonant type and above resonance type. Various constructions of vibrating screens and the tuning of their technological parameters are discussed in [7,8,9,10,11]. The resonance regime is desirable; however, its control is complicated due to vulnerability of the sieving process to changes in bulk material thickness, particle distribution over the deck and their physical properties [12,13]. Moreover, resonance frequencies can be harmful for screen construction. Even in the above resonate solutions, most of the damages occur during startup and stopping procedures, when the device is passing through the resonance frequency. Most vibrating screens use inertial vibrating exciters, which are designed with respect to the specified trajectory (linear, circular, elliptical) and direction of the sieve movement [14]. Such trajectories can be realized with one electric motor and various numbers of unbalances that rotate synchronously using forced kinematic or dynamic synchronization [15]. Different means of kinematic synchronization can be used, e.g., gears [16], belt transmissions or elastic links with nonlinear stiffness [17]. Industrial applications and various research use two-mass systems [18] or systems with different numbers of vibrators and unbalanced masses [19]. Nowadays, most popular solutions are based on drives with two vibrators, providing synchronous rotation of unbalanced masses. Parameters of the trajectory can be controlled by changes in the direction, frequency or phases of the vibrators [20]. Another popular solution uses three or four independently installed vibrators and frequency control during synchronization [21,22]. The research reported in this article focuses on the application of a semi-active element—a magnetorheological damper (MR damper)—in the suspension of a vibrating screen with only one vibrator.

The MR damper consists of a piston and a cylinder which is filled with an MR fluid. The MR fluid is made of magnetizable particles suspended in carrier fluid, e.g., minerals [23]. These particles are subjected to a magnetic field induced by electric coils, which are located in the vicinity of piston gaps, reorganize in a chain-like structure and increase the damping parameter of the MR damper on a macroscopic scale. MR dampers, as an example of a semi-active system, are favored over active solutions for their low energy consumption. They are widely used for vibration control in mountain bikes [24], automotive applications and all-terrain vehicles [25]; for improvement of driving safety [26]; in the construction machinery [27]; and in buildings and bridges [28].

The behavior of MR dampers is commonly analyzed based on characteristics of generated force with respect to axial piston velocity. Here, increased complexity can be justified by the force saturation exhibited for higher piston velocities or hysteresis loops, as presented in [29]. Thus, controlling of the MR damper force is challenging, and it commonly requires preliminary identification of at least a limited MR damper model in order to apply an open-loop of the closed-loop force control approach. Methods of modeling MR dampers are generally divided into phenomenological, input-output and behavioral models. Phenomenological models reflect the internal design of an MR damper and try to describe the occurring phenomena, e.g., by taking into account velocity profiles of MR fluid flowing through a piston gap, as presented in [30]. Additionally, the yield shear stress or magnetic field distribution can be analyzed as reported in [31] for an unbalanced rigid rotor damped by dedicated MR films. More demanding transient analysis of MR damper operation can utilize methods of computational fluid dynamics or distributed modeling of the fluid–structure interaction. Such methods discussed, e.g., in [32,33], can additionally take into account the influences of thermal properties of applied materials or fluid cavitation.

Semi-active vibration control applications which utilize model-based control approaches generally require the MR damper models to exhibit limited computational complexity in order to be easily implemented in a real-time controller. Thus, combinations of input–output and behavioral models are mostly used. The Bingham model presented in [34] consists of two dominant components, i.e., Coulomb friction and viscous damping. Further, the Gamota–Filisko model presented in [35] includes Bingham, Kelvin–Voight and Hooke body models which are connected in series. The well-known Bouc–Wen model [36] of an MR damper and its extension, the Spencer [35] model, are favored for their compact form and ability to map dominant nonlinear features of MR damper behavior. The following should be mentioned: force saturation greenwhich is revealed for higher piston velocities and hysteresis loops greenwhich are noticeable in force–velocity characteristics. Additionally, an input–output modeling approach can be applied based on the hyperbolic tangent function, as presented in [37], which is favored for low computational complexity, and simultaneously, it allows for good adjustment to the shape of force–velocity characteristics.

**Figure 1:**
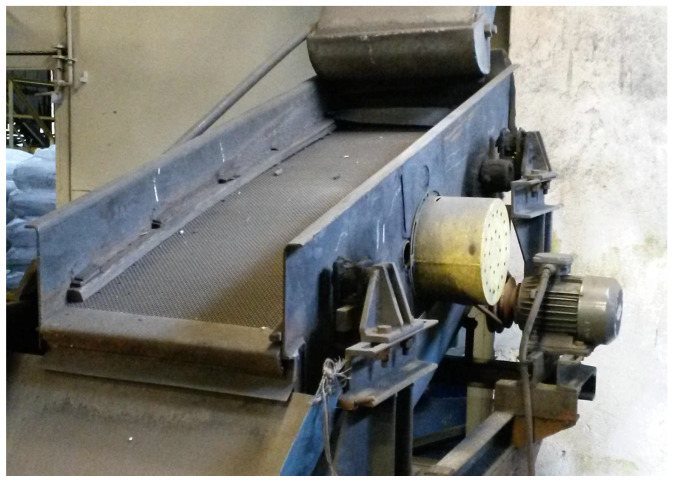
A semi-industrial vibrating screen with a single mechanical exciter based on rotating unbalanced mass.

To make the research concerning vibrating screen modifications more efficient, many researchers use modeling and simulation techniques at the first stage of research. There are reports of this approach being applied for circular vibrating screens [38,39], linear vibrating screens [40,41,42], an elliptical vibrating screen [43] and banana screens [44,45]. Models are developed for operational diagnosis [46], for diagnosis of spring failures [47] or for vibration exciter operation analysis [48]. The model of a vibrating screen we created was verified with the measurement data from experiments on a semi-industrial vibrating screen (see Figure 1). The model was than expanded with the Bouc–Wen model of the MR damper, identified during previous research based on measurement data obtained from an MTS (material testing system). In the case of identification of both the vibrating screen model and the MR damper model, the methods of analysis of vibration measurements are extremely important [49]. In the case of the screen, additional effects deteriorating the quality of measurements need to be taken into account, e.g., impulsive noise generated by processed material and methods for filtering of such [50].

The research reported in this article used modeling and simulation techniques to show the possibility of changing and controlling the vibrating screen’s trajectory using the semi-active element of its suspension. Various algorithms for trajectory control are well-known in the literature and were applied for various applications. Apart from the classical PID (proportional-integral-derivative) controller, the adaptive backstepping method accompanied with a Lyapunov function was used for trajectory tracking algorithm of an automated guided vehicle in [51]. An MPC (model predictive control) including a novel Hammerstein model was used for controlling the gimbal system mounted on an unmanned aerial vehicle under external disturbances, as presented in [52]. An LTV-MPC (linear time-varying model predictive control) was used for trajectory tracking control for an autonomous vehicle in [53]. However, in order to simplify the trajectory control algorithm which is proposed in this article and developed for future implementation in the real-time controller of the screen suspension, a straight-forward control method was applied. It is intended for emulation of force generated by an additional virtual vibrator. Changing the relationship between the work of the virtual and the real vibrator allows for modification of the vibration trajectory.

The majority of studies presented in the literature dedicated to screen suspension with variable parameters exhibit limited possibilities for adaptation. It is time-consuming to adjust a screen to varying process parameters, making it almost impossible to achieve fast control of vibration trajectory during screen operation. Applications of MR dampers in screen suspension are known in the literature; however, they do not have a control system and they are limited to operate in open-loop configuration or even mostly using a constant control current supplying the MR dampers. A design of MR damper dedicated to a vibrating screen, described in [54], was applied in order to enhance the screening efficiency. Other authors carried out experiments for different screening amplitudes and constant control currents to the MR damper. The key results showed the influences of these parameters on the variance of vibration displacement and screening efficiency. Another study presented in [55] and dedicated to the MR damper installed in a vibrating screen and showed a trispectrum. Correlation dimension analysis was similarly carried out for different constant control currents only. That analysis was extended in [56], where preliminary experiments were carried out for a single MR damper. Further, it was indicated that constant control current can be used for mitigation of extensive vibrations occurring during the start-up and stopping phases of the screen’s operation. Relative to the studies currently available in the literature, the main contributions of the approach proposed in this paper are as follows:

- We propose an application with several MR damper models oriented horizontally and vertically and located in the front and rear parts of the vibrating screen model, which allows for efficient vibration control;
- The proposed solution allows for online modification of the vibration trajectory by emulation of force generated by a virtual and additional mechanical exciter;
- Application of MR dampers in the vibrating screen allows faster transitions through the resonant frequencies on startup and stop procedures;
- The Bouc–Wen model was adjusted for a range of vibration frequencies that spans up to 20 Hz, which allows for the application of a single MR damper model during all phases of screen operation;
- The dissipative domain of the MR damper was evaluated based on the Bouc–Wen model, which allowed simplification of the model in implementation and simulation;
- The developed methodology for identifying the screen model is scalable, and it can be applied for future vibrating screens of different physical sizes and for validation of vibration control algorithms.

The article consists of five sections. In Section 2, a dynamic model of the presented vibrating screen is defined. Section 3 presents experimental results obtained for the actual industrial screen and reports results of identification of a dynamic model of the actual screen. Furthermore, a Bouc–Wen model of MR damper is defined and the procedure of Bouc–Wen model identification is presented based on experimental results obtained for harmonic excitation. Section 4 proposes a novel trajectory control approach which is applied to the implemented screen model. The proposed control algorithm is further validated and discussed for different configurations and desired vibration trajectories. Finally, the conclusions are presented in Section 5.

## 2. Dynamic Model of Vibrating Screen

**Figure 2:**
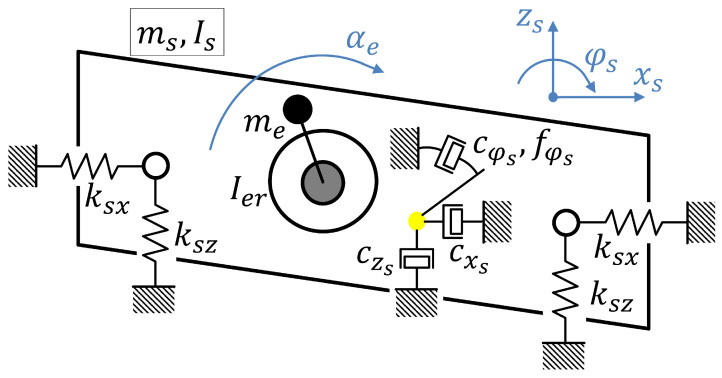
Mechanical representation of the dynamic model of vibrating screen with a single mechanical exciter.

The considered dynamic model of a screen was implemented and applied in the presented study as a two-dimensional mechanical structure of lumped parameters. A mechanical representation of the discussed model is presented in Figure 2. A mechanical exciter with unbalanced masses driven by a single electric motor is a source of screen vibration. For the purpose of the presented study, the lateral movement of the screen was neglected, since both the construction and unbalanced masses of the exciter are symmetrically located with respect to the longitudinal axis of the screen.

### 2.1. Structure of the Dynamic Model

The dynamic screen model consists of a rigid body corresponding to the riddle of the screen, including a sieve, which is supported on a viscoelastic suspension. The considered model takes into account the influence of the gravitational field, and it exhibits four DOFs (degrees of freedom) overall. Three DOFs describe the motion of the screen riddle, and they are denoted as $x_{s}$, $z_{s}$ and $\varphi_{s}$. The first two mentioned DOFs correspond to horizontal $x_{s}$ and vertical $z_{s}$ displacements of the riddle’s center of mass, whose absolute location is defined as ($x_{s}$, $z_{s}$). Variable $\varphi_{s}$ corresponds to the third DOF, and it describes the pitch motion of the riddle. The riddle, including the sieve, which is the key vibrating part of the screen, is characterized by its mass and moment of inertia denoted as $m_{s}$ and $I_{s}$, respectively. For the purpose of further analysis, $\dot{x}$ and $\ddot{x}$ are defined as first and second derivatives of a selected variable *x*, respectively. Additionally, horizontal and vertical directions are denoted as *x* and *z*, respectively.

The assumed model of screen suspension mainly consists of horizontally and vertically oriented linear springs. Here, stiffness parameters are assumed as the same for both front and rear suspension parts depending if the horizontal or vertical direction is considered, and they are denoted as $k_{sx}$ or $k_{sz}$, respectively. These springs are attached to the selected points of the front and rear parts of the riddle. Thus, coordinates describing locations of these points are denoted as ($x_{sf}$, $z_{sf}$) and ($x_{sr}$, $z_{sr}$), respectively. These locations are defined based on $x_{s}$, $z_{s}$ and $\varphi_{s}$ and with respect to the frame of reference associated with the stationary screen base. They additionally depend on parameters ($l_{sf}$, $h_{sf}$) and ($l_{sr}$, $h_{sr}$) describing locations of the suspension attachment points with respect to the riddle’s center of mass and its frame of reference. Additionally, damping related mainly to the suspension is described by parameters of horizontal and vertical viscous damping acting on the riddle center of mass, denoted as $c_{x_{s}}$ and $c_{z_{s}}$, respectively. The rotation of the riddle is influenced by parameters of rotary viscous damping $c_{\varphi_{s}}$ and dry friction $f_{\varphi_{s}}$. As a result, it was assumed that the above-mentioned parameters are sufficient to map the dominant behavior of the actual suspension.

The mechanical exciter with unbalanced masses is an additional component independent of the riddle driven by a electric-motor-related torque denoted as $M_{m}$. The electric motor is fixed to the screen base, and the unbalanced masses are mounted on both sides of a shaft which is attached to the screen riddle in lateral direction with the possibility of rotation. The rotating shaft is connected to the electric motor using a rubber belt. The proportion between the diameters of motor shaft and unbalanced-masses-related shaft is denoted as $p_{e}$ (details about physical parameters of the considered semi-industrial vibrating screen are presented in Section 3). Thus, a torque applied to the unbalanced masses can be defined as $M_{e}=p_{e}\cdot M_{m}$.

Consequently, the fourth DOF, denoted as angle $\alpha_{e}$, describes instantaneous position of these unbalanced masses, i.e., their relative inclinations with respect to the longitudinal axis of the riddle. Furthermore, the relationship between the angular velocity of the motor and the unbalanced-masses shaft ${\dot{\alpha }}_{e}$ can be defined as ${\dot{\alpha }}_{m}=p_{e}\cdot \left({\dot{\alpha }}_{e}+{\dot{\varphi }}_{s}\right)$. The unbalanced masses are described with its overall mass $m_{e}$ and moment of inertia $I_{er}$ evaluated with respect to the axis of its rotation. The exciter is attached to the riddle using a rotating member at location defined by ($x_{er}$, $z_{er}$) with respect to the screen base. The imbalance of this structure is described by a distance denoted as $r_{e}$ and defined as being from the axis of rotation to the center of mass of the exciter $m_{e}$. Thus, location of $m_{e}$ with respect to the stationary screen base is defined as ($x_{em}$, $z_{em}$) which depends on all above-mentioned DOFs, $r_{e}$ and parameters ($l_{er}$, $h_{er}$) describing the location of the exciter rotation axis with respect to the screen riddle.

Operation of the electric motor and its torque $M_{m}$, which depends on the instantaneous motor slip *s*, was described by taking torque characteristics generated by a typical actual asynchronous electric motor. The considered motor exhibits a synchronous angular velocity ${\dot{\alpha }}_{m0}$, nominal torque $M_{n}$ and nominal slip $s_{n}$ evaluated for the nominal angular velocity denoted as ${\dot{\alpha }}_{mn}$. The typical torque characteristics include additional amplification of torque for angular velocities close to zero, i.e., a starting torque denoted as $M_{a}$ apart from the motor’s maximum torque denoted as $M_{k}$ and corresponding motor slip $s_{k}$. Such operation of an electric motor can be mathematically described as a composition of different torque characteristics, as suggested in [57]. Each of these characteristics can be evaluated based on the Kloss equation recalled in [58]. Thus, for the presented study, the composition of switchable Kloss equations evaluated for different values of parameters was implemented as follows:

(1) $${\overset{´}{M}}_{m}\left(s\right)=\left\{\begin{array}{ccc} 2M_{a}{\left(s+\frac{1}{s}\right)}^{-1} & \mathrm{for} & {\dot{\alpha }}_{m}<{\dot{\alpha }}_{m,th}\\ 2M_{k}{\left(\frac{s}{s_{k}}+\frac{s_{k}}{s}\right)}^{-1} & \mathrm{for} & {\dot{\alpha }}_{m}\geq {\dot{\alpha }}_{m,th} \end{array}\right.,$$

where a threshold angular velocity denoted as ${\dot{\alpha }}_{m,th}$ indicates an angular velocity of intersection of constituent characteristics described by the Kloss equation. The torque of the final model of the electric motor $M_{m}={\overset{´}{M}}_{m}-f_{\alpha_{m}}$ additionally maps its rotating resistance using a parameter of rotary dry friction $f_{\alpha_{m}}$.

### 2.2. Mathematical Description of the Screen Model

Generalized coordinates describing the screen model were adopted for the purpose of the Euler–Lagrange equations analogously to the definition of the DOFs, i.e., $q_{k}\in \left\{x_{s},z_{s},\varphi_{s},\alpha_{e}\right\}$. Kinetic *T* and potential *V* energies are defined for the considered screen model as follows:

(2) $$\begin{array}{cc} T= & \frac{1}{2}m_{s}{\dot{x}}_{s}^{2}+\frac{1}{2}m_{s}{\dot{z}}_{s}^{2}+\frac{1}{2}I_{s}{\dot{\varphi }}_{s}^{2}+\frac{1}{2}m_{e}{\dot{x}}_{em}^{2}+\frac{1}{2}m_{e}{\dot{z}}_{em}^{2}+\frac{1}{2}I_{em}{\left({\dot{\varphi }}_{s}+{\dot{\alpha }}_{e}\right)}^{2},\\ V= & \frac{1}{2}k{\left(x_{sf}-x_{sf0}\right)}^{2}+\frac{1}{2}k{\left(z_{sf}-z_{sf0}\right)}^{2}+\frac{1}{2}k{\left(x_{sr}-x_{sr0}\right)}^{2}+\frac{1}{2}k{\left(z_{sr}-z_{sr0}\right)}^{2}\\ \; & +m_{s}gz_{s}+m_{e}gz_{em}, \end{array}$$

where *g* denotes gravitational acceleration and $I_{em}$ denotes the moment of inertia defined with respect to the center of exciter mass $m_{e}$ located at ($x_{em}$, $z_{em}$). It can be evaluated using inverted Steiner’s theorem about parallel axis as $I_{em}=I_{er}-m_{e}r_{e}^{2}$. Here, initial locations of the front and rear parts of the screen suspension and related to its stiffness are denoted as ($x_{sf0}$, $z_{sf0}$) and ($x_{sr0}$, $z_{sr0}$), respectively.

A set of non-conservative forces, defined as $F_{i}\in \left\{F_{cx_{s}},F_{cz_{s}},M_{c\varphi_{s}},M_{e}\right\}$, acts on the corresponding points of the screen along the virtual displacements $r_{i}\in \left\{x_{s},z_{s},\varphi_{s},\varphi_{e}\right\}$. Symbols $F_{cx_{s}}$, $F_{cz_{s}}$ and $M_{c\varphi_{s}}$ correspond to viscous damping forces and a torque related mainly to the screen suspension, acting on the riddle’s center of mass in horizontal, vertical and angular directions, respectively. The generalized forces $Q_{k}\in \left\{Q_{cx},Q_{cz},Q_{c\varphi },Q_{c\alpha }\right\}$ are evaluated depending on virtual displacements $r_{i}$, non-conservative forces $F_{i}$ and generalized coordinates $q_{i}$ as follows:

(3) $$\begin{array}{cc} \; & Q_{cx}=F_{cx_{s}},\\ \; & Q_{cz}=F_{cz_{s}},\\ \; & Q_{c\varphi }=M_{c\varphi_{s}}+M_{e},\\ \; & Q_{c\alpha }=M_{e}. \end{array}$$

The mathematical description of the considered screen model was evaluated based on Euler–Lagrange equations and resulted in the following four ordinary differential equations:

(4) $$\begin{array}{cc} m_{s}{\ddot{x}}_{s}= & -m_{e}{\ddot{x}}_{em}+F_{sx},\\ m_{s}{\ddot{z}}_{s}= & -m_{e}{\ddot{z}}_{em}+F_{sz}+F_{gsz},\\ I_{s}{\ddot{\varphi }}_{s}= & -m_{e}{\ddot{x}}_{em}\left(h_{er}\cos\varphi_{s}-l_{er}\sin\varphi_{s}\right)\\ \; & +m_{e}{\ddot{z}}_{em}\left(h_{er}\sin\varphi_{s}+l_{er}\cos\varphi_{s}\right)+M_{s}+M_{gs},\\ I_{er}{\ddot{\alpha }}_{e}= & -m_{e}{\ddot{x}}_{er}r_{e}\sin\left(\alpha_{e}+\varphi_{s}\right)-m_{e}{\ddot{z}}_{er}r_{e}\cos\left(\alpha_{e}+\varphi_{s}\right)-I_{er}{\ddot{\varphi }}_{s}+M_{e}+M_{ge}. \end{array}$$

Interactions of the screen with its suspension (horizontal $F_{sx}$ and vertical $F_{sz}$ forces, torque $M_{s}$) and the influence of the gravitational field (vertical force $F_{gsz}$ and torques related to the riddle $M_{gs}$ and the mechanical exciter $M_{ge}$) were intentionally generalized in Equations (4). That allowed us to emphasize the internal dynamics of the dynamic screen model and to simplify further model implementations. These variables are derived as follows:

(5) $$\begin{array}{cc} F_{sx}= & F_{kfx}+F_{krx}+F_{cx_{s}},\\ F_{sz}= & F_{kfz}+F_{krz}+F_{cz_{s}},\\ F_{gsz}= & -m_{s}g-m_{e}g,\\ M_{s}= & F_{kfx}\cdot \left(z_{sf}-z_{s}\right)+F_{krx}\cdot \left(z_{sr}-z_{s}\right)\\ \; & -F_{kfz}\cdot \left(x_{sf}-x_{s}\right)-F_{krz}\cdot \left(x_{sr}-x_{s}\right)+M_{c\varphi_{s}},\\ M_{gs}= & -m_{e}g\left(x_{er}-x_{s}\right),\\ M_{ge}= & -m_{e}g\cdot r_{e}\cos\left(\alpha_{e}+\varphi_{s}\right). \end{array}$$

Forces generated by the horizontal and vertical stiffness components of the front and rear screen suspension are denoted as $F_{kfx}$, $F_{kfz}$ and $F_{krx}$, $F_{krz}$, respectively. Horizontal $F_{cx_{s}}$ and vertical $F_{cz_{s}}$ viscous damping forces depend on parameters $c_{x_{s}}$ and $c_{z_{s}}$, respectively. The damping torque $M_{c\varphi_{s}}$ depends on parameters of viscous damping $c_{\varphi_{s}}$ and dry friction $f_{\varphi_{s}}$.

### 2.3. Implementation of the Vibrating Screen Simulator

The vibrating screen simulator was implemented based on the ordinary differential equations presented in (4) which were reformulated into the following nonlinear matrix state-space form:

(6) $$\dot{X}=F_{A}\left(X,U\right).$$

The simulated system was described using 8 state variables assumed as a list of generalized coordinates and their derivatives, which are defined in the form of a vector $X={\left[x_{s},z_{s},\varphi_{s},\alpha_{e},{\dot{x}}_{s},{\dot{z}}_{s},{\dot{\varphi }}_{s},\dot{\alpha_{e}}\right]}^{T}$. The vector $F_{A}={\left[f_{x_{s}},f_{z_{s}},f_{\varphi_{s}},f_{\alpha_{e}},{\dot{x}}_{s},{\dot{z}}_{s},{\dot{\varphi }}_{s},\dot{\alpha_{e}}\right]}^{T}$ consists of nonlinear functions of *X* and *U*, and for some elements, it depends directly on other state variables. The external variables listed in the vector *U* correspond to the following forces and torques: $M_{e}$ and those related to interaction with the suspension or gravitational field, $F_{sx}$, $F_{sz}$, $F_{gsz}$, $M_{s}$, $M_{gs}$ and $M_{ge}$, which are defined in Equation (5).

The screen model was implemented using a Matlab environment. The state-space matrix equation, Equation (6), was solved during simulation for a desired simulation time using the ode45 function implemented in Matlab environment, i.e., a fifth-order Runge–Kutta method with a variable integration step. However, it requires reformulation of differential Equations (4), which are nonlinear and complex, to the form of nonlinear functions included in the state-space equation, Equation (6). For this purpose, equations related to ${\ddot{x}}_{s}$, ${\ddot{z}}_{s}$ and ${\ddot{\varphi }}_{s}$ were initially grouped and reformulated into the following form:

(7) $$D\left(X\right)\cdot {\dot{X}}_{1}=F_{P}\left(X,U\right),$$

where $X_{1}={\left[{\dot{x}}_{s},{\dot{z}}_{s},{\dot{\varphi }}_{s}\right]}^{T}$ denotes a vector of selected state variables. Symmetrical matrix D(X) depends on nonlinear terms of state variables *X*; a vector $F_{P}$ consists of nonlinear functions of *X* and *U*.

Equation (7) can be solved analytically mainly by inversion of matrix D(X), which allows for direct calculation of second derivatives of generalized coordinates included in ${\dot{X}}_{1}$. However, such an analytical derivation could introduce redundant terms which are difficult to notice and reduce, and it can consequently make further implementation more challenging. Thus, a compromise method was decided on to calculate the inversion of D(X) in every simulation step after the substituting of known instantaneous values of state variables and checking the rank of matrix D(X). Finally, taking advantage of calculated variables ${\dot{X}}_{1}$ for a selected simulation step, the $\ddot{\alpha_{e}}$ can be obtained by reformulation of the corresponding differential Equation (4).

### 2.4. Procedure of Modeling and Identification of the Vibrating Screen Model

**Figure 3:**
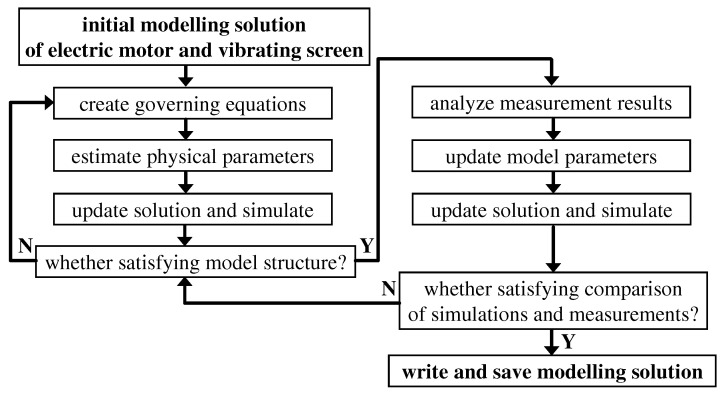
Flowchart for the procedure of modeling and identification of the vibrating screen model.

The process of the screen modeling and identification was divided into two main phases, as presented in Figure 3, related to defining the model’s structure and final tuning of the model’s parameters. First, an initial modeling solution of the electric motor and the vibrating screen was assumed. Further, governing equations were created describing the model’s structure. Physical parameters of the model were estimated within the first phase based on observations and physical dimensions of the actual screen design. The updated model was simulated and its response analyzed in order to assess whether the model’s structure was satisfying.

The second phase was based on observation of measurements, which allowed for further tuning of the model’s parameters. Consequently, the screen model was updated and simulated. Validation of the model was based on several quantities describing screen motion, which are presented in the following forms: time diagrams of three degrees of freedom: $x_{s}$, $z_{s}$ and $\varphi_{s}$; and trajectories of vibration displacement and acceleration evaluated for a selected riddle part. Comparison of results obtained for simulations and measurements was used for assessment of whether the model conformed to the measurements to a sufficient degree or whether the model needed further parameter tuning either modification of its structure. Finally, the resultant screen was saved and applied for further analysis of trajectory control algorithm.

## 3. Identification of Semi-Industrial Vibrating Screen and Magnetorheological Damper

This study is dedicated to the industrial single deck screen presented in Figure 1. The experimental screen was equipped with a single mechanical exciter with unbalanced masses generating a circular trajectory of the riddle vibration. The presented experimental setup is dedicated to the development and validation of a vibration control algorithm desired for improvement of the sieving process. The presented screen was intended to have future modifications to its suspension in order to include semi-active dampers. Such dampers allow for adaptation of the suspension’s characteristics during screen operation to production and sieving needs. Future research will take into account applications of MR dampers whose damping parameters can be changed in milliseconds.

The first phase of the study presented in the current manuscript was deriving and validating a screen simulator, including a model of MR damper behavior. The evaluated screen simulator described in the current section needs to map dominant characteristics of the actual screen. It allows one to make the developed control algorithm be representative of an actual screen. Values of screen parameters estimated in the further part of the current section are presented in Table 1, and notation of the model’s parameters conforms to what was defined in Section 2. The second phase of the research reported in next sections was dedicated to the development and validation of the proposed semi-active control algorithm of trajectory control using the implemented screen simulator.

### 3.1. Physical Dimensions and Estimation of Selected Parameters

The riddle of the experimental screen was 46 cm wide and 1.26 m long, and it was inclined to the horizontal side by about 18${}^{\circ }$. Side walls of the riddle were 38 cm high at the front and 40 cm high at the rear. The riddle was mostly made of 6-mm-thick sheet metal. Additionally, three metal pipes were located transversely to the riddle in the vicinity of the mechanical exciter, which were intended to connect the side walls of the riddle with each other. The middle pipe was also used to guide the rotating shaft, which connected the unbalanced masses of the exciter located on both sides of the screen. The rotary axis of the mechanical exciter was located close to the estimated center of mass of the riddle at a distance $l_{er}$ = 0.011 m horizontally and $h_{er}$ = 0.048 m vertically, with respect to the side plane of the screen.

The physical dimensions of the riddle and screen structure were used for estimations of selected parameters required for the screen simulator. The mass of the riddle, which was the key vibrating object of the analyzed system, was obtained as $m_{s}$ = 102.6 kg assuming the density of steel ρ = 7900 kgm${}^{-3}$, and the riddle’s moment of inertia defined for a transverse axis of rotation through the center of mass was estimated as $I_{s}$ = 14.78 kgm${}^{2}$. Parameters of the mechanical exciter, which was partly attached to the riddle, were not included in $m_{s}$ and $I_{s}$.

Unbalanced masses of the mechanical exciter were half-circle shaped of radius equal to 10 cm and thickness equal to 22 mm. Apart from unbalanced masses, physical parameters of the pulley and exciter shaft needed to be taken into account. As a result, the overall mass of the mechanical rotating part of the exciter was estimated as $m_{e}$ = 32.4 kg, and the corresponding moment of inertia defined with respect to the axis of rotation was equal to $I_{er}$ = 0.14 kgm${}^{2}$. For the given value of $m_{e}$, and based on geometrical properties of unbalanced masses, the distance between the center of mass and axis of rotation was calculated as $r_{e}$ = 7 mm.

The mechanical vibration exciter was driven by a 3-phase electric motor with $P_{m}$ = 0.75 kW designed for continuous operation. The proportion between diameters of the motor shaft and exciter shaft was $p_{e}$ = 1.25. The applied motor exhibited 4 poles, and consequently synchronous angular velocity was equal to ${\dot{\alpha }}_{m0}$/2 π = 1500 rpm = 25 Hz for frequency of the mains electricity of 50 Hz. Nominal angular velocity of the motor was equal to ${\dot{\alpha }}_{mn}$/2 π = 1400 rpm = 23.3 Hz, which gives a nominal motor slip of $s_{n}$ = 0.067. Consequently, the nominal torque of the motor can be calculated as $M_{n}=P_{m}{\dot{\alpha }}_{mn}$ = 5.12 Nm.

**Figure 4:**
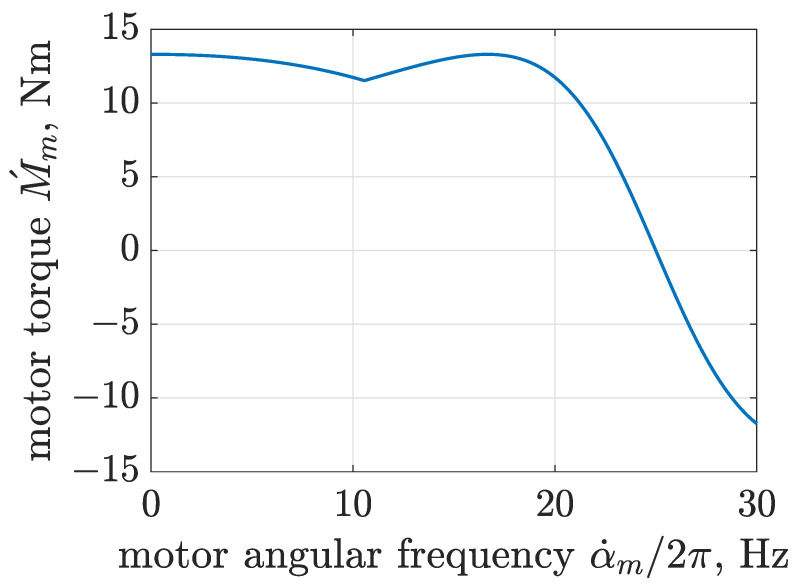
Characteristics of the electric motor model based on switchable Kloss equations.

According to the characteristics of electric motors of a similar class, the maximum motor torque was assumed as $M_{k}$ = 2.6 $M_{n}$ = 13.31 Nm, and the starting torque was assumed as $M_{a}$ = 2.4 $M_{n}$ = 12.29 Nm. Thus, the motor slip related to the maximum torque $M_{k}$ was evaluated as $s_{k}$ = 0.333, and consequently, the threshold angular velocity defined in Section 2 was calculated as ${\dot{\alpha }}_{m,th}$ = 2 π 10.57 rads${}^{-1}$. The characteristics of the applied model of the electric motor can be evaluated using Figure 4.

The modeled electric motor operates in motoring mode for angular frequencies from 0 to 25 Hz (${\dot{\alpha }}_{m0}$), and it exhibits positive torque within this frequency range. For angular frequencies greater than 25 Hz, the motor starts to operate as a power generator, in which case the motor torque takes a negative value. The characteristics show two maxima for motor torques corresponding to $M_{a}$ and $M_{k}$. The presented approach of motor modeling allows one to map influence of rotating unbalanced masses on angular velocity of the motor. Instantaneous rotation of the mechanical exciter or varying weight and parameters of the sieved material has an influence on the angular velocity and phase of the electric motor, as in the case of the actual screen.

### 3.2. Experimental Setup and Identification Experiments

**Figure 5:**
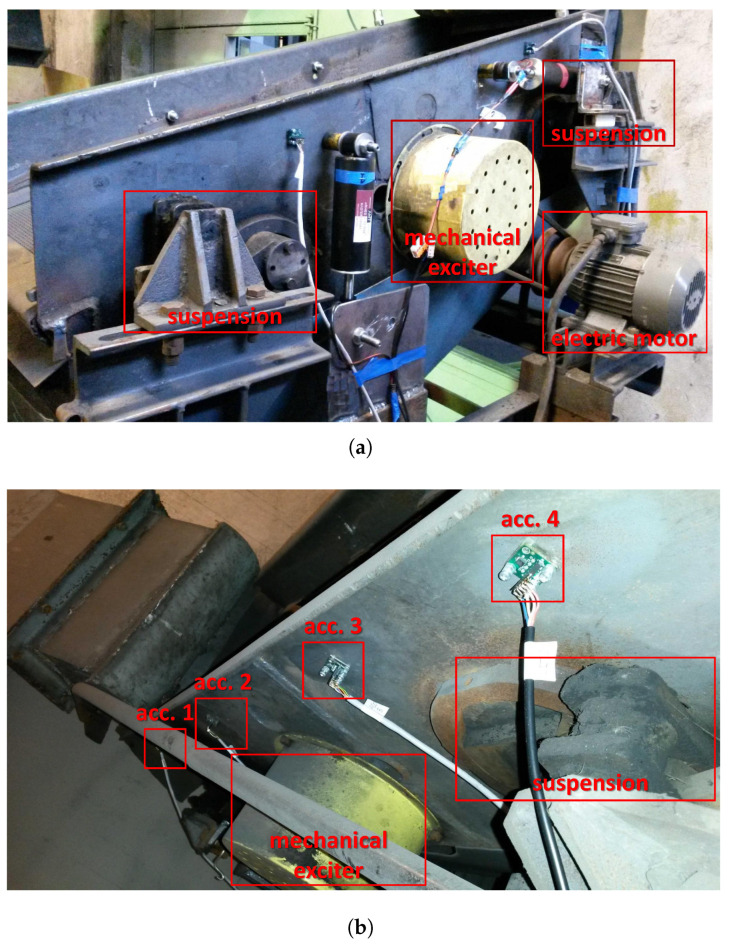
Measurement system applied for identification of screen model: (**a**) mechanical structure of the screen, (**b**) accelerometers attached to the sidewall of the screen riddle at the measurement points.

The vibrating screen configured for experiments is presented in Figure 5a, where main components are marked, i.e., the screen suspension and mechanical exciter driven by the electric motor. Vibration of the experimental screen, as presented in Figure 5b, was measured using an MEMS (micro-electro-mechanical system) with 3-axis accelerometers of type ADXL325 and ADXL326 produced by Analog Devices, which exhibit measurement ranges of ±5 g or ±16 g, respectively. Acceleration was assessed by measurement of deflection of an internal moving mass suspended by polysilicon springs within the sensor. The measurement of deflection was carried out using differential capacitors fixed to the moving mass. The resultant measurement signal, which was proportional to acceleration, was conditioned by internal circuits and fed outside the sensor in the form of analog output voltage. Voltage of the output signal for both types of accelerometers could vary within the nominal range from about 0.5 to 2.5 volts; the nominal offset value is equal to 1.5 volts.

In the case of accelerometer axes denoted as *X* and *Y*, which are parallel to the case of the integrated circuit, the nominal frequency bandwidth of measurements was equal to 1600 Hz, and in the case of the sensor’s vertical axis denoted as *Z*, the frequency bandwidth was equal to 550 Hz. The former *X* and *Y* axes of each applied accelerometer were used during the considered measurements of screen vibration. Standard evaluation boards dedicated to applied accelerometers were used during experiments, which allowed for robust assembly of sensors in the screen’s structure. Each evaluation board additionally included several capacitors, which in combination with resistors built into the sensor integrated circuit, formed a first-order analog antialiasing filter of cutoff frequency equal to 50 Hz.

Four accelerometers were used for experiments, and they were located in the left sidewalls of the screen riddle, as presented in Figure 5b. The locations of the consecutive sensors were defined by their corresponding horizontal and vertical distances in meters from the center or riddle mass as follows: (−0.501, 0.304), (−0.191, 0.185), (0.339, 0.015) and (0.559, −0.035). These locations allowed for comprehensive analysis of the vibration trajectories of different parts of the riddle and the sieve in a plane defined by the vertical and longitudinal axes of the screen. Measurement signals generated by the sensors and filtered using the antialiasing filter were acquired by a dedicated measurement system with a sampling frequency equal to $f_{s}=1/T_{s}$ = 1000 Hz and preprocessed for further analysis, including conversion of physical units and compensation of signal offset. Velocities and displacements of selected parts of the vibrating screen were estimated based on acceleration measurements by single or double integration with inertia, respectively. An example of a displacement estimation is presented by the following formula:

(8) $$z\left(i\right)=H_{b}\left(z^{-1}\right)\cdot {\left(\frac{T_{s}}{1-z^{-1}}\right)}^{2}\cdot a\left(i\right),$$

where *i* denotes number of a sample of a selected signal. Displacement, velocity and acceleration digital signals are denoted in the presented study as *z*, *v* and *a*, respectively. Acceleration measurements taken from the experimental setup with vibrating screen were twice numerically integrated in order to estimate displacement signals. These signals were further processed by additional discrete-time Butterworth filter denoted as $H_{b}\left(z^{-1}\right)$ defined based on an unit delay operator denoted as $z^{-1}$. Depending on analysis of processed measurement signals:

- the lowpass filter was used for fine processing of acceleration with cut-off frequency of 50 Hz;
- the highpass filter with cut-off frequency equal to 4 Hz was used for filtering of estimated displacement signals dedicated to time-domain analysis;
- the highpass filter of cut-off frequency 18 Hz was used for signals dedicated to the analysis of vibration trajectory.

### 3.3. Evaluation of Parameters of the Dynamic Screen Model

Vibration of the screen was tracked by the selected *j*-th sensor measuring acceleration in horizontal and vertical directions, which can be described as follows:

(9) $$\begin{array}{cc} \; & {\ddot{x}}_{j}={\ddot{x}}_{s}-h_{j}\left({\dot{\varphi }}_{s}^{2}\sin\varphi_{s}-{\ddot{\varphi }}_{s}\cos\varphi_{s}\right)-l_{j}\left({\dot{\varphi }}_{s}^{2}\cos\varphi_{s}+{\ddot{\varphi }}_{s}\sin\varphi_{s}\right),\\ \; & {\ddot{z}}_{j}={\ddot{z}}_{s}-h_{j}\left({\dot{\varphi }}_{s}^{2}\cos\varphi_{s}+{\ddot{\varphi }}_{s}\sin\varphi_{s}\right)+l_{j}\left({\dot{\varphi }}_{s}^{2}\sin\varphi_{s}-{\ddot{\varphi }}_{s}\cos\varphi_{s}\right). \end{array}$$

Evaluation of parameters of the dynamic screen model was performed based on acceleration evaluated independently in horizontal and vertical direction as averages over available accelerometers and denoted as ${\ddot{x}}_{a}$ and ${\ddot{z}}_{a}$, respectively. The model validation was also carried out based on estimation of the riddle inclination angle $\varphi_{s}$. According to Equation (9), which is linear with respect to $l_{i}$ and $h_{i}$, the above-mentioned averaged acceleration is equivalent to acceleration measured in location as an average of all locations of sensors as follows: $l_{a}=1/4\sum_{j=1}^{4}l_{j}$ = 0.051 m, $h_{a}=1/4\sum_{j=1}^{4}h_{j}$ = 0.117 m. The $\varphi_{s}$ angle was evaluated based on displacement signals estimated for accelerometers located in the extreme positions in the front and rear parts of the screen riddle according to the following:

(10) $$\varphi_{s}=\arcsin\frac{\left(h_{1}-h_{4}\right)\left(x_{1}-x_{4}\right)}{{\left(l_{1}-l_{4}\right)}^{2}+{\left(h_{1}-h_{4}\right)}^{2}}.$$

**Figure 6:**
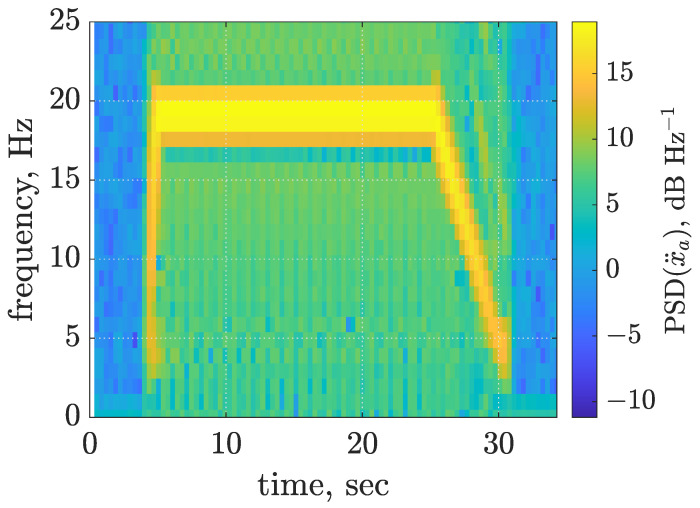
Time–frequency characteristics of the averaged horizontal acceleration of the screen riddle based on measurements.

Each series of the experiment consisted of three phases of operation of the vibrating screen: start-up, operation with nominal vibration frequency and stopping the mechanical exciter. Time–frequency characteristics evaluated for the averaged horizontal acceleration of the screen riddle are presented in Figure 6. The angular frequency of the mechanical exciter about 1 s after start-up of the electric motor reached the steady-state frequency of about 19.2 Hz. The mechanical exciters rotated with the nominal frequency during 20 s of nominal operation. Finally, the electric motor was turned off, and consequently, the rotating shaft with unbalanced masses began to slow down, making the screen vibrate at successive, decreasing frequencies, which lasted about 5 s.

**Figure 7:**
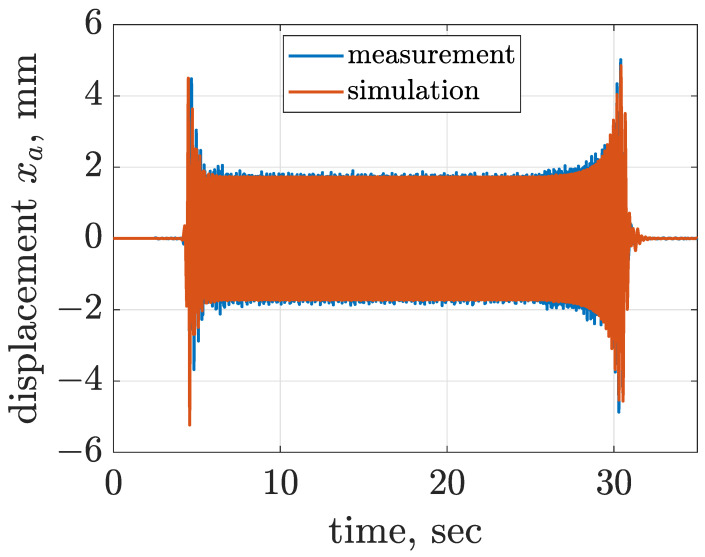
Comparison of time diagrams of horizontal displacement $x_{a}$ corresponding to the averaged sensor location evaluated for measurement and simulation results.

**Figure 8:**
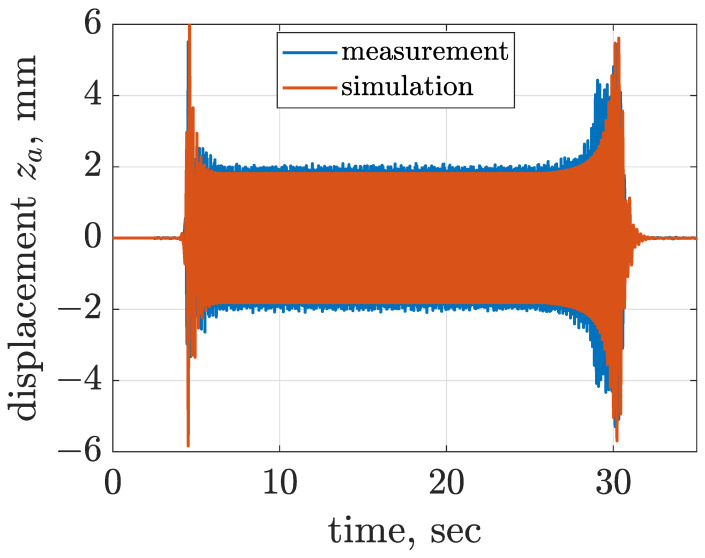
Comparison of time diagrams of vertical displacement $z_{a}$ corresponding to the averaged sensor location evaluated for measurement and simulation results.

**Figure 9:**
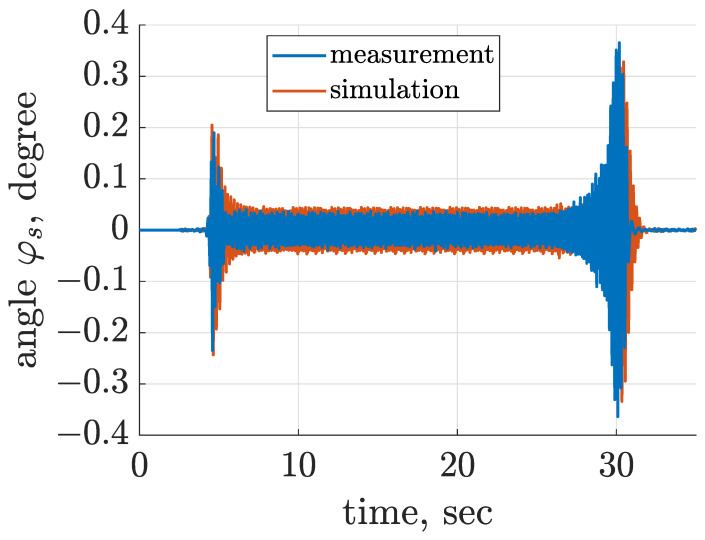
Comparison of time diagrams of riddle angle $\varphi_{s}$ evaluated for measurement and simulation results based on acceleration signals for locations at extreme front and rear measurement points of the vibrating riddle.

The considered screen is over-resonant, which means that during start-up and stopping the angular frequency of the mechanical exciter for a moment coincides with the resonance frequency of the screen. Estimated horizontal and vertical displacements of the screen riddle are presented in Figure 7 and Figure 8, where occurrence of resonance is clearly visible for increasing amplitude of vibration at close to 4.5 Hz, slightly dependent on the direction of vibration. Furthermore, it can be noticed that the amplitude of vibration displacement is approximately equal to 1.8 mm during the nominal phase of screen operation. The third degree of freedom $\varphi_{s}$ estimated according to Equation (10) is presented in Figure 9. The time diagram of $\varphi_{s}$ shows that for the start-up phase, the amplitude reached 0.2${}^{\circ }$, and for the stopping phase the maximum value of amplitude was close to 0.4${}^{\circ }$ at the moment of resonance. During the nominal operation of the screen, $\varphi_{s}$ oscillated within an amplitude close to 0.07${}^{\circ }$. Despite the fact that maximum amplitudes of $\varphi_{s}$ seem to be low, it should be noticed that $\varphi_{s}$ = 0.4${}^{\circ }$ resulted in an approximately 2 mm change in vertical displacement of the front suspension $z_{sf}$, which was about 25% of the overall displacement $z_{sf}$. Thus, the influence of $\varphi_{s}$ on the response of the dynamical screen model was significant in this study.

The remaining parameters of the screen model, i.e., stiffness and damping parameters of the screen suspension, and the rotational friction of the electric motor, were evaluated by comparison of simulation results and measurements, which gave satisfactory results, as presented in Figure 7, Figure 8 and Figure 9. The suspension of the screen consisted of four parts, each attached to the riddle on one side and to the screen base on the other side. Points of suspension-to-riddle attachment were at distance $l_{sf}$ = 0.34 m horizontally and $h_{sf}$ = 0.14 m vertically at the riddle front and at distance $l_{sr}$ = 0.35 m horizontally and $h_{sr}$ = 0.08 m vertically at the riddle rear. A single part of the screen suspension was designed in the form of a mechanism which consisted of two members and three rotary joints, and the last joint built into the riddle was coupled with a rotary spring.

**Figure 10:**
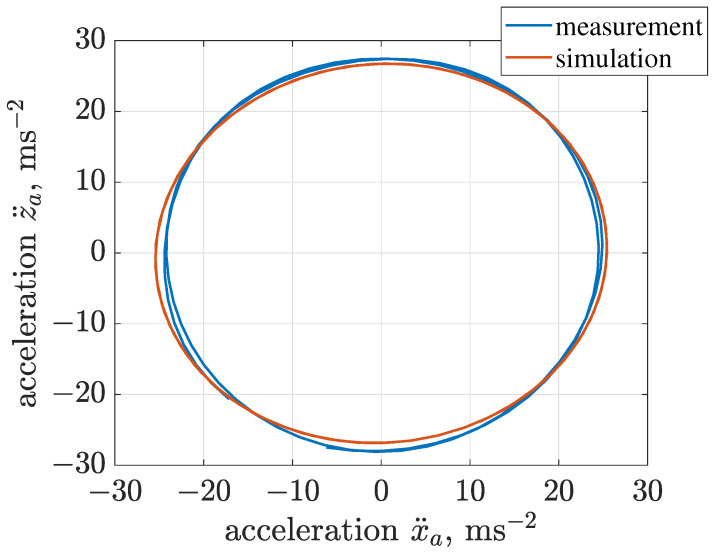
Comparison of trajectories of screen vibration acceleration evaluated for measurement and simulation results.

**Figure 11:**
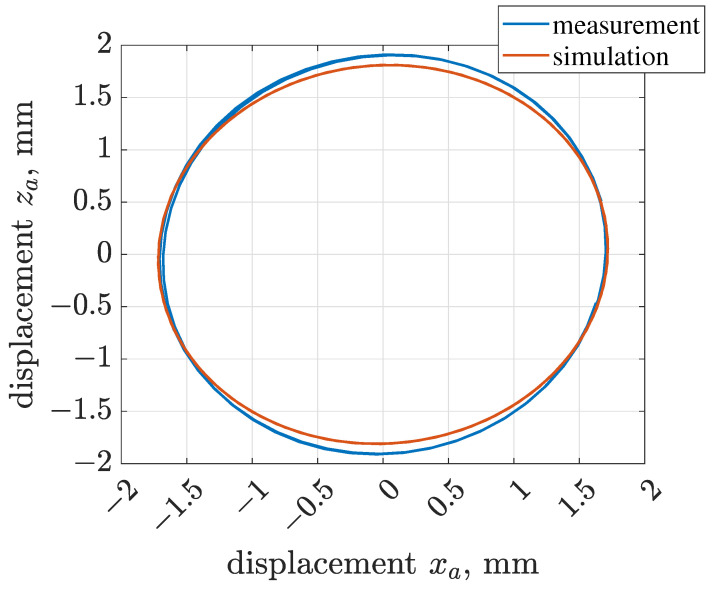
Comparison of trajectories of screen vibration displacement evaluated for measurement and simulation results.

The equivalent horizontal and vertical stiffness levels of front and rear suspensions were initially assumed based on the mass of the vibrating riddle and measured resonance frequencies, and further tuned which gave the following assumptions: $k_{sx}$ = 44,966 Nm${}^{-1}$ and $k_{sz}$ = 57,094 Nm${}^{-1}$. Subsequently, damping parameters related to the screen suspension were tuned as follows: $c_{x_{s}}$ = 800 Nsm${}^{-1}$, $c_{z_{s}}$ = 800 Nsm${}^{-1}$, $c_{\varphi_{s}}$ = 31 Nms rad${}^{-1}$ and $f_{\varphi_{s}}$ = 1.4 Nm. Rotational friction of the electric motor was selected as $f_{\alpha_{m}}$ = 2.24 Nm. The implemented model of the vibration screen was additionally validated by comparing trajectories of vibration acceleration and displacement obtained for experiments and simulations in Figure 10 and Figure 11.

Amplitudes of trajectories of vibration acceleration for both simulations and experiments were close to 25 ms${}^{-2}$. Furthermore, we confirmed that the single mechanical exciter generated riddle vibration trajectory of a circular shape. Commonly, it can be stated that the vibration trajectories of the screen riddle present a phase shift between its horizontal $x_{a}$ and vertical $z_{a}$ displacement. Thus, $x_{a}$ and $z_{a}$, which are in-phase results in linear trajectory, and $x_{a}$ and $z_{a}$, shifted in phase by π/2, correspond to the circular and elliptical trajectories. The presented study relates the synthesis and validation of the trajectory control. Thus, the compatibility of the model with the real object is crucial for the presented application when analyzing the vibration trajectory obtained for nominal screen operation. The significant compatibility of simulation and experimental results justifies the estimates of the parameters of the implemented screen model, which are reported in Table 1, including the parameters of the Bouc–Wen model of the MR damper.

### 3.4. Non-Dimensional Frequency-Domain Analysis

**Figure 12:**
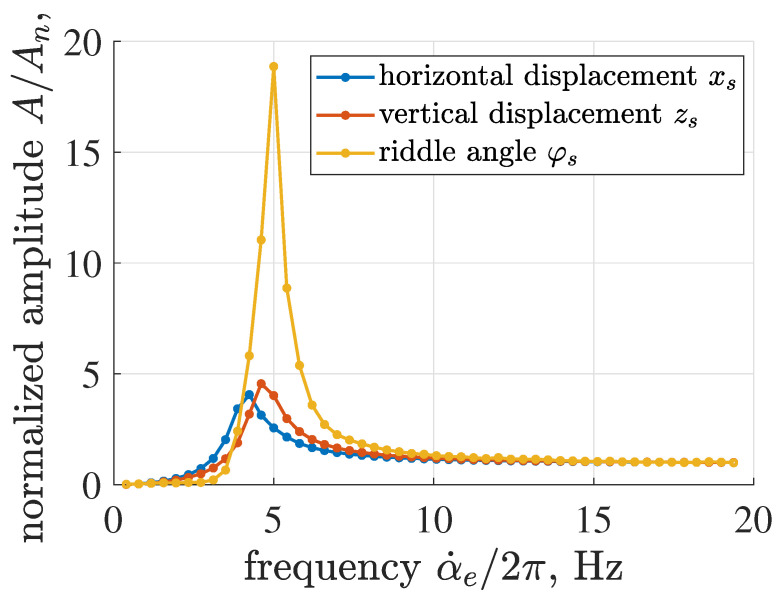
Comparison of non-dimensional normalized amplitudes of horizontal, vertical and angular motion of the vibrating riddle evaluated for the identified screen model in the frequency domain.

Additional analysis of a non-dimensional normalized amplitude of vibration obtained for the identified screen model was carried out in the frequency domain for three degrees of freedom, i.e., $x_{s}$, $z_{s}$ and $\varphi_{s}$, as presented in Figure 12. Consecutive points *j* marked in the characteristics were evaluated in steady-state for the corresponding constant angular frequency of the mechanical exciter ${\dot{\alpha }}_{e,j}$. Each value of amplitude *A* was calculated as a root mean squared value of the selected signal within five cycles of its harmonic response. Further, all amplitude values obtained for the selected degree of freedom were normalized by a nominal vibration amplitude denoted as $A_{n}$. The value of $A_{n}$ corresponds to the nominal frequency of screen operation of 19.2 Hz.

The time diagrams can be divided into sub-resonant, resonance and over-resonant ranges. The sub-resonant frequency range is defined up to 4.2–5 Hz, where the normalized amplitude is relatively low. Further, it can be noticed that resonance frequencies are slightly different for corresponding degrees of freedom, i.e., 4.2 Hz for $x_{s}$, 4.6 Hz for $z_{s}$ and 5 Hz for $\varphi_{s}$. The amplitudes of resonance are similar for $x_{s}$ and $z_{s}$—close to 4.0 and 4.5, respectively. The resonance peak of $\varphi_{s}$ is significantly greater, which is clearly visible regarding the measurements presented in Figure 9, and approximately equal to 18.9. The vibrating screen is subjected to vibration generated by the mechanical exciter, whose amplitude is proportional to the square of its angular frequency. Thus, despite the increasing damping exhibited by the implemented mechanical model, the amplitudes of all degrees of freedom stabilize at a constant value.

### 3.5. Bouc–Wen Model of the Magnetorheological Damper

**Figure 13:**
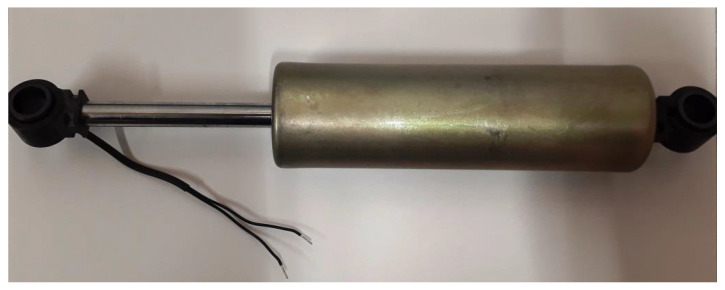
Mechanical structure of a magnetorheological damper, type RD-8041-1, manufactured by Lord Corporation.

Magnetorheological dampers manufactured by Lord Corporation of type RD-8041-1 presented in Figure 13 were selected for further simulation tests. This type of MR damper exhibits piston stroke equal to 74 mm, and it is appropriate for vibration control in various applications, from industrial machines to road and off-road vehicles.

According to the corresponding documentation, these MR dampers are recommended to be continuously controlled by electric currents of up to 1 ampere. Thus, further analysis of the MR damper’s behavior was focused on the above-mentioned level of control current. The well-known Bouc–Wen model of MR dampers was applied for mapping dominant nonlinear features of MR damper behavior, i.e., force saturation revealed for higher piston velocities and hysteresis loops indicated in force–velocity characteristics. The key part of the Bouc–Wen is the nonlinear differential equation, which can be defined as follows:

(11) $$\begin{array}{cc} \; & {\dot{p}}_{bw}=-\alpha_{bw}\cdot |v_{mr}|\cdot p_{bw}\cdot |p_{bw}{|}^{n_{bw}-1}-\beta_{bw}\cdot v_{mr}\cdot {\left|p_{bw}\right|}^{n_{bw}}+A_{bw}\cdot v_{mr},\\ \; & F_{bw}=-\gamma_{bw}p_{bw}-c_{bw}v_{mr}, \end{array}$$

where $v_{mr}$ denotes the axial velocity of the damper’s piston; $p_{bw}$ denotes the displacement of the Bouc–Wen model, which is included in the final formula for force $F_{bw}$ generated by the Bouc–Wen model. $\alpha_{bw}$, $\beta_{bw}$, $A_{bw}$, $n_{bw}$, $\gamma_{bw}$ and $c_{bw}$ denote parameters of the model which need to be estimated based on results obtained from an identification experiment.

### 3.6. Identification of the MR Damper Model

Identification experiments of the MR damper were carried out using a material testing system. The examined MR damper was subjected to axial sinusoidal kinematic excitation at different amplitudes and frequencies. During the experiments, the MR damper was supplied by a control current signal consisting of unit steps of different values generated independently by a dedicated control system. Three configurations were selected for identification experiments, which were characterized by the following amplitudes and frequencies: (1.5 Hz, 15 mm), (6 Hz, 5 mm) and (20 Hz, 2 mm). The range of excitation frequencies was selected intentionally in order to cover the range of frequencies reached during all phases of operation of the screen and its mechanical exciter. Similarly, the selected displacement amplitudes are compatible with those reached by the considered industrial screen.

**Figure 14:**
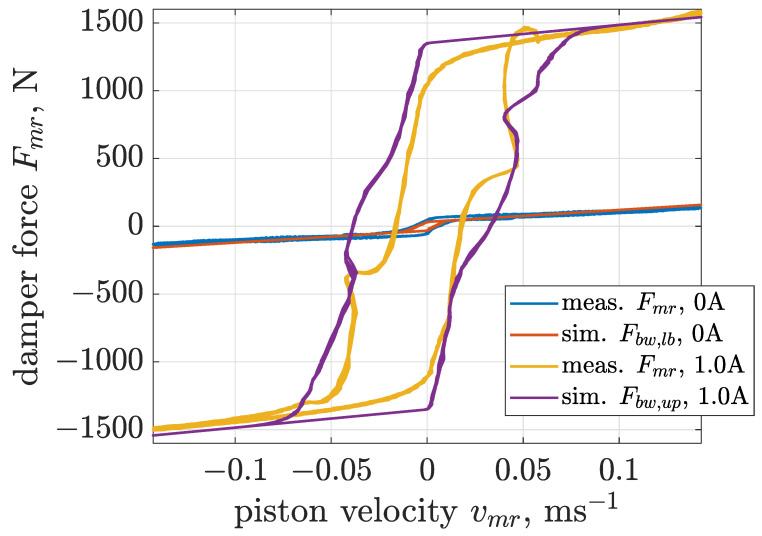
Comparison of force–velocity characteristics obtained for the MR damper and the Bouc–Wen model subjected to the sinusoidal excitation of frequency 1.5 Hz and displacement amplitude 15 mm.

**Figure 15:**
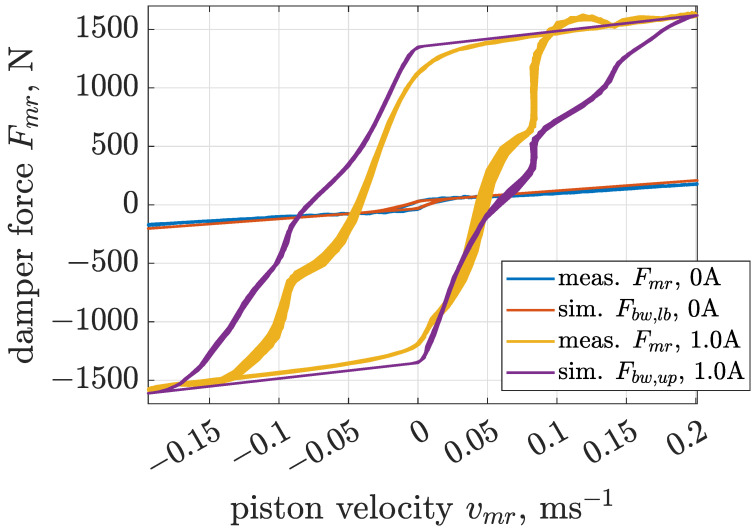
Comparison of force–velocity characteristics obtained for the MR damper and the Bouc–Wen model subjected to the sinusoidal excitation of frequency 6 Hz and displacement amplitude 5 mm.

**Figure 16:**
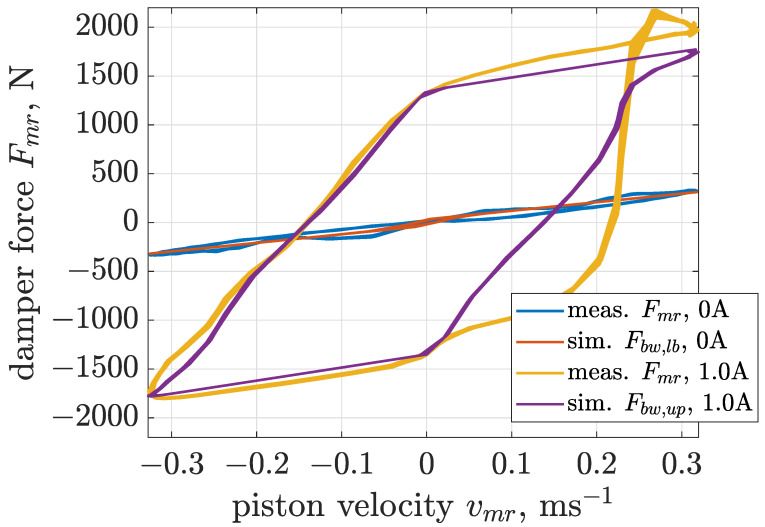
Comparison of force–velocity characteristics obtained for the MR damper and the Bouc–Wen model subjected to the sinusoidal excitation of frequency 20 Hz and displacement amplitude 2 mm.

Estimation of Bouc–Wen parameters for a selected value of control current was carried out based on a cost function defined as the mean squared error calculated between measurements and the outputs of the model simultaneously for all three cases of different excitation frequencies. The next part of the presented analysis focuses on boundary models of the MR damper. Thus, Figure 14, Figure 15 and Figure 16 present comparisons of measurements and model responses evaluated for control currents equal to 0 and 1 amperes. Estimated parameters of applied Bouc–Wen models are listed in Table 1.

A characteristic feature of MR damper behavior is significant dependence of the sizes of the hysteresis loop and force amplitudes indicated in the force–velocity curves on sinusoidal excitation frequencies. The applied Bouc–Wen model allows one to map this dependency for the whole range of analyzed excitation frequencies. This advantage is crucial for the considered screen, as comprehensive analysis of its operation requires a single and accurate model of the MR damper which is valid for all operating conditions of the screen.

The hysteresis loop of the MR damper can be described using different approaches, such as a static function of a certain quantity describing the piston motion or a dynamic model of the signal path defined from the piston motion to the output damper force. Some research proposed modeling the velocity–force hysteretic behavior using a first order linear filter is presented in [29]. Thus, similarly to the case of circular trajectory of the riddle vibration discussed previously, the hysteresis loop of the MR damper can be emulated for other studies by the phase shift between signals of the piston velocity $v_{mr}$ and the damper force $F_{mr}$.

## 4. Trajectory Control Applied for the Screen Dynamic Model

**Figure 17:**
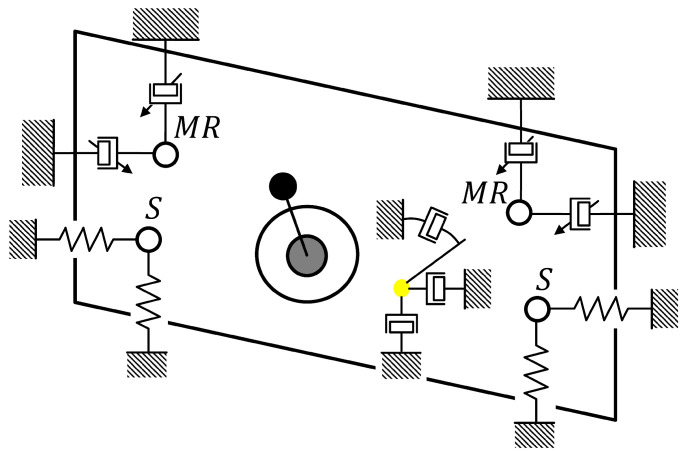
Mechanical representation of the dynamic model of the vibrating screen, including the Bouc–Wen model of the MR damper.

The features of screen vibration trajectory are crucial for efficiency of sieving process. Moreover, possibility of adaptation of vibration trajectory to varying parameters of the production process and processed material is favored in modern industry. Such adaptability can be introduced into the standard design of the vibrating screen by application of MR dampers in its suspension, as presented in Figure 17. The MR dampers, as semi-active dampers of one type, are favored over active solutions for their low energy-consumption.

In this study, MR dampers controlled in an appropriate manner were intended to modify the vibration trajectory to a desired shape. Furthermore, in the case of screens which are equipped with only a single mechanical exciter generating a circular vibration trajectory, adaptive control of screen suspension could be the only way to generate a linear vibration trajectory.

### 4.1. Simulation Results of Passive Suspension

Initial tests of the screen model, including Bouc–Wen models, were carried out assuming constant control current supplying the MR dampers. A similar test procedure consisted of three phases, i.e., start-up and stopping of the mechanical exciter and nominal operation of the vibrating screen. Three configurations of screen suspension were validated: the suspension without an MR damper included, and those with Bouc–Wen models $F_{bw,lb}$ and $F_{bw,ub}$ corresponding to control current equal to 0 and 1 ampere, respectively, as presented in Section 3.6.

**Figure 18:**
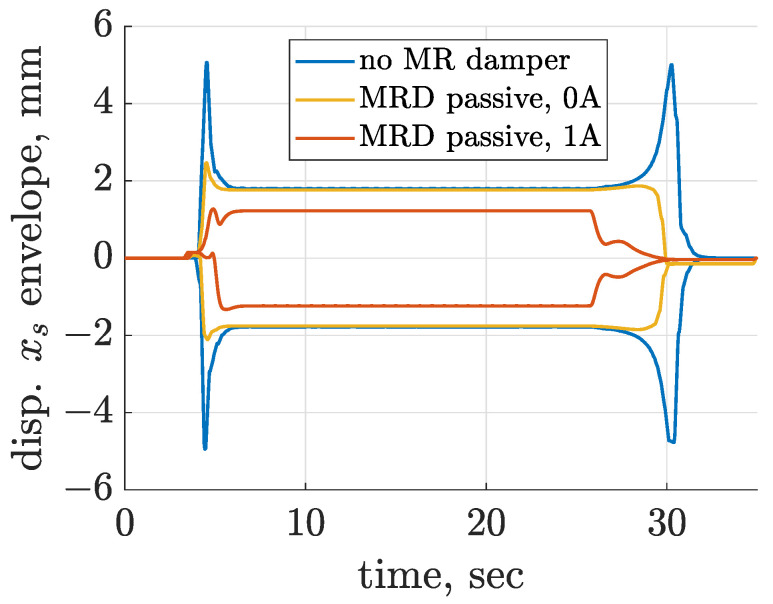
Comparison of time diagrams of envelopes evaluated for the horizontal displacement $x_{s}$ in the case of the screen model without an MR damper model included and with an MR damper controlled by a constant current equal to 0 or 1 ampere.

The envelopes of horizontal displacement $x_{s}$ of the center of riddle mass were plotted in Figure 18 for the above-mentioned cases. The first envelope is analogous to time-diagrams presented in Figure 7, where occurrence of resonance is clearly visible for the start-up and stopping phases. However, it can be noticed that including the MR damper model supplied by zero control current significantly mitigated resonant peaks, and at the same time it left the vibration amplitude unchanged for the nominal phase of operation.

**Figure 19:**
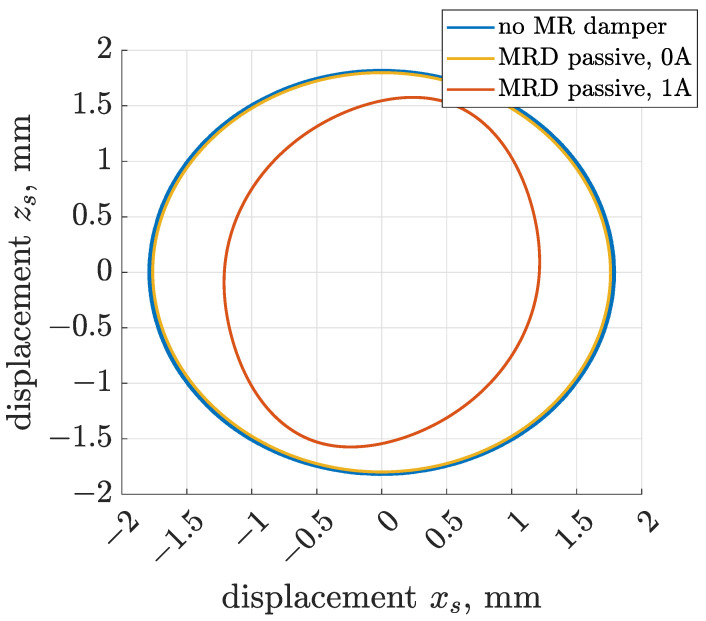
Comparison of trajectories of screen vibration displacement evaluated for the screen model without an MR damper model included and with an MR damper controlled by a constant current equal to 0 or 1 ampere.

The increase in MR damper control current from 0 to 1 ampere significantly influenced vibration amplitude in all phases of screen operation. In the case of the stopping phase, vibration was mitigated clearly faster as soon as the electric motor shut down. The amplitude of vibration for the nominal vibration frequency was significantly decreased from 1.8 to 1.2 and 1.6 mm for the *x* and *z* directions, respectively, as presented in Figure 19. The difference between the displacement trajectories obtained for 0 and 1 ampere and the influence of greater control currents are noticeable in Figure 19.

This analysis related to passive suspension can be compared to those of other studies available in the literature related to the application of an MR damper for a vibrating screen. Despite the fact that different studies have used slightly different constructions of screens and MR dampers, a valuable qualitative comparison could still be carried out. A study presented by the other authors [56] showed a difference in the damping of the resonance tested for three similar configurations with or without an MR damper in the vibrating screen. The examined vibration screen exhibited an amplitude of vibration for nominal excitation frequency close to 4 mm, which is twice that applied in the current research. However, it was similarly shown in time diagrams of vibration signal that the energized MR damper allowed for significant resonance damping.

Another study presented in [54] discussed the application of a manufactured and tested MR damper whose operation in a vibrating screen was analyzed in frequency and time domains. Different vibration amplitudes were applied, from 3–5 mm, and vibration frequency up to 16 Hz was used. It was also shown that the variance of vibration displacement decreased for increasing MR damper control current. In conclusion, MR dampers included in the screen suspension allows one to modify the amplitude of vibration and consequently the size of the vibration trajectory; however, its circular shape remains the same, independently of the constant control current, when using a single mechanical exciter.

### 4.2. Description of the Control Algorithm

**Figure 20:**
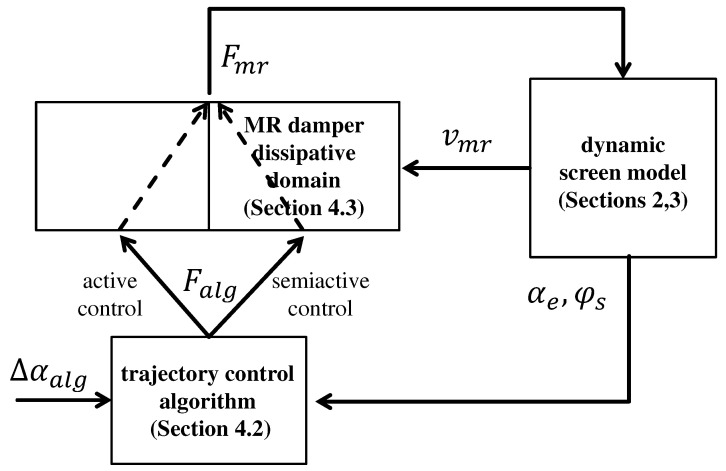
Implemented control block diagram, including the dynamic model of the vibrating screen, the trajectory control algorithm and the MR damper dissipative domain.

The proposed control block diagram presented in Figure 20 includes three components, i.e., a dynamic model of the vibrating screen described in Section 2 and Section 3, the trajectory control algorithm presented in Section 4.2 and an implementation of semiactive control based on the MR damper dissipative domain defined in Section 4.3.

In the case of the implemented screen simulator, it was assumed that the MR damper Bouc–Wen model interacts with the screen model in predefined locations which are compatible with those intended for future experiments planned for the considered industrial screen. Thus, it was assumed that single MR dampers were fixed horizontally and vertically to the front and rear parts of the screen riddle in locations defined as follows: $l_{mrf}$ = 0.229, $h_{mrf}$ = 0.040, $l_{mrr}$ = −0.111 and $h_{mrr}$ = 0.169 m, respectively. Consequently, forces denoted as $F_{mr,i}$ generated by four MR dampers included in the implemented model contribute to the resultant balance of forces and moments of forces, which influence the dynamics of the screen.

The goal of the control algorithm is to emulate the force generated by a virtual and additional mechanical exciter which rotates in the opposite direction to the real exciter with an additional phase shift denoted as $\Delta \alpha_{alg}$. The opposite rotation of two mechanical exciters allows for the generation of a linear vibration trajectory. Assuming that a force vector of approximately $F_{e}=m_{e}{\left({\dot{\alpha }}_{e}+{\dot{\varphi }}_{s}\right)}^{2}r_{e}$ is generated by the real exciter in a direction dependent on angle $\varphi_{e}=\alpha_{e}+\varphi_{s}$, the fictitious exciter rotating opposite would be described horizontally and vertically as follows:

(12) $$\begin{array}{cc} \; & F_{alg,x}=-1/N_{mr,x}\cdot F_{e}\cos\left(-\alpha_{e}+\varphi_{s}+\Delta \alpha_{alg}\right),\\ \; & F_{alg,z}=1/N_{mr,z}\cdot F_{e}\sin\left(-\alpha_{e}+\varphi_{s}+\Delta \alpha_{alg}\right). \end{array}$$

Forces $F_{alg,x}$ and $F_{alg,z}$ are further distributed into separate MR damper models oriented horizontally or vertically, indicated by $N_{mr,x}$ or $N_{mr,z}$, respectively.

### 4.3. Dissipative Domain of the MR Damper Model

Industrial or automotive applications with MR dampers require implementation of a damper force control algorithm. Commonly, an open-loop approach to force control is applied based on an inverse MR damper model, since installation of force sensors in such applications often significantly increases costs and weakens the structure of the device.

A typical approach to the simulation research of semi-active control is to apply an inverse model which is fully compatible with the MR damper model. It comes down to a case when desired force generated by the vibration control algorithm can be directly led to the vibrating model after being limited to a region of reachable force generated by the MR damper for instantaneous conditions. The region of reachable forces is called the dissipative domain of an MR damper, and it can be defined as group of damping characteristics obtained for control currents generated within their acceptable ranges.

**Figure 21:**
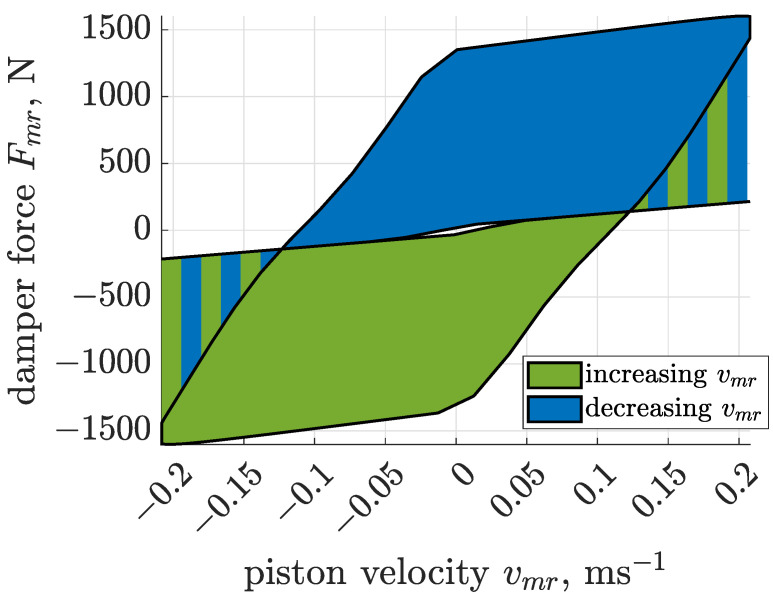
Dissipative domain generated based on the response of the identified Bouc–Wen model, subjected to the sinusoidal displacement excitation of frequency 18.85 Hz and an amplitude of 1.8 mm.

For the purpose of the presented study, a dissipative domain was generated as presented in Figure 21, based on the response of the identified Bouc–Wen model. The model was subjected to a sinusoidal excitation of frequency 18.85 Hz and an amplitude 1.8 mm, which are related to nominal conditions of screen operation.

The dissipative domain was defined as a region of reachable forces located between damping characteristics related control currents of 0 and 1.0 ampere. It should be noticed that different force regions are reachable for increasing and decreasing piston velocities. As a result, force generated by the MR damper model is based on limited force $F_{alg}$ generated by the control algorithms, as follows:

(13) $$F_{mr}=\left\{F_{alg}:\min\left(F_{bw,lb},F_{bw,ub}\right)<F_{alg}<\max\left(F_{bw,lb},F_{bw,ub}\right)\right\},$$

where $F_{bw,lb}$ and $F_{bw,ub}$ denote force dependent on damper piston velocity $v_{mr}$ generated by the Bouc–Wen model according to Equation (11) and for a set of its parameters related to lower lb or upper bounds up, respectively, listed in Table 1.

### 4.4. Simulation and Discussion of the Screen Trajectory Control

Analysis of vibration trajectory control was divided into two stages. Firstly, the implemented simulation environment was validated by application of active control without utilizing the dissipative domain, as presented in the control block diagram in Figure 20. Secondly, the dissipative domain was activated and the trajectory control algorithm was tested for the target semi-active configuration related to MR damper behavior.

**Figure 22:**
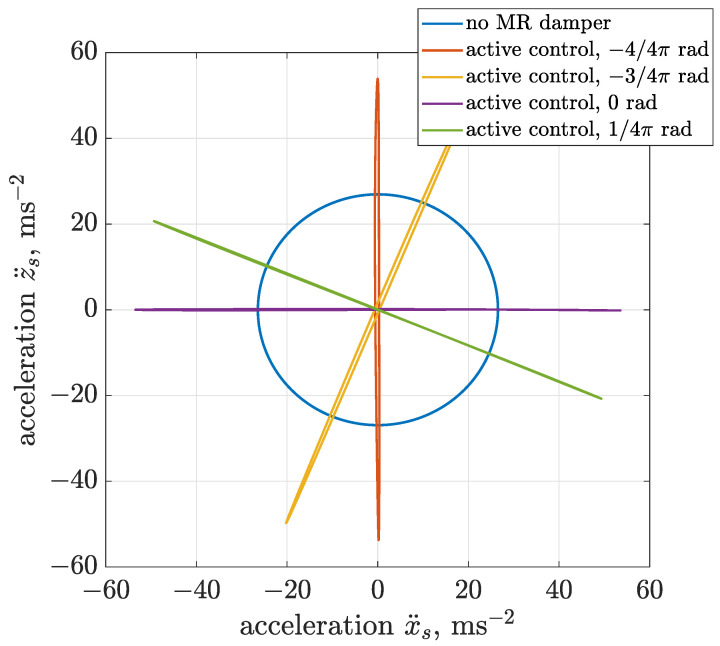
Different configurations of phase shift $\Delta \alpha_{alg}$ dedicated to active generation of linear trajectories of vibration acceleration.

**Figure 23:**
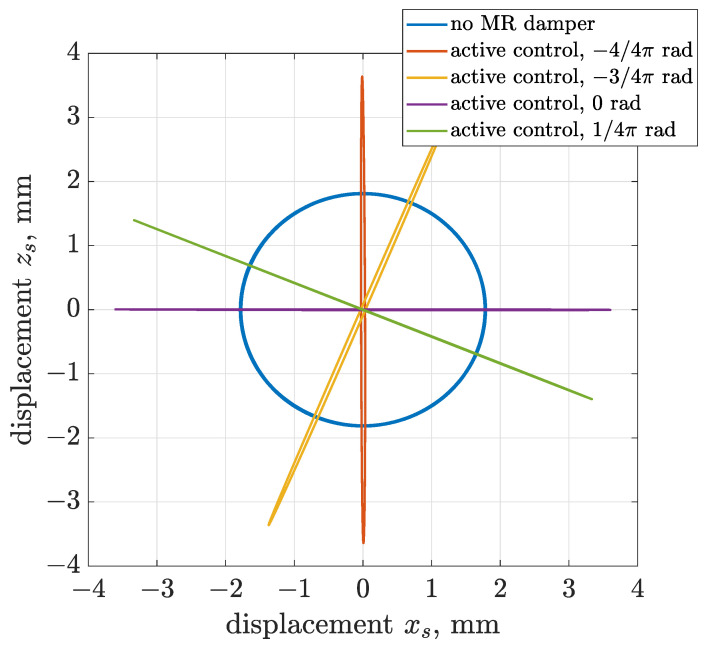
Different configurations of phase shift $\Delta \alpha_{alg}$ dedicated to active generation of linear trajectories of vibration displacement.

Results obtained for the active control are presented in the form of trajectories of vibration acceleration and displacement in Figure 22 and Figure 23. The presented simulation cases were obtained for the following values of algorithm phase shifts $\Delta \alpha_{alg}$—0, 1/4 π, 4/4 π and 5/4 π—and compared with the case of a vibrating screen operating without an MR damper.

It can be seen based on acceleration and displacement trajectories that the proposed control algorithm successfully modifies the shape of the trajectory form circular to linear. The slope of the trajectory can be freely changed from horizontal to vertical or in between. It should be also noticed that the amplitudes of acceleration and displacement doubled, since an active approach of force generation is applied, which adds vibration energy to the considered system.

**Figure 24:**
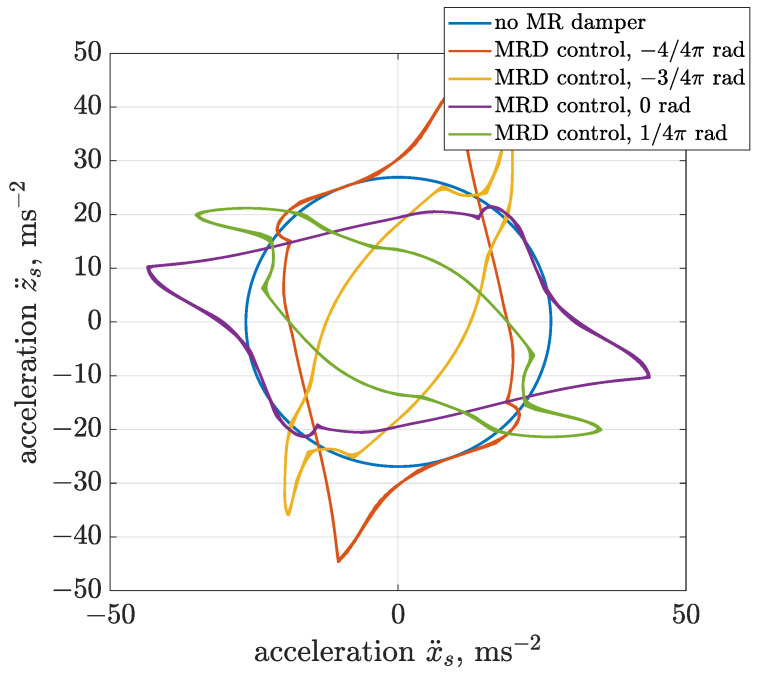
Different configurations of phase shift $\Delta \alpha_{alg}$ dedicated to semi-active generation of linear trajectories of vibration acceleration using the dissipative domain of the Bouc–Wen MR damper model.

**Figure 25:**
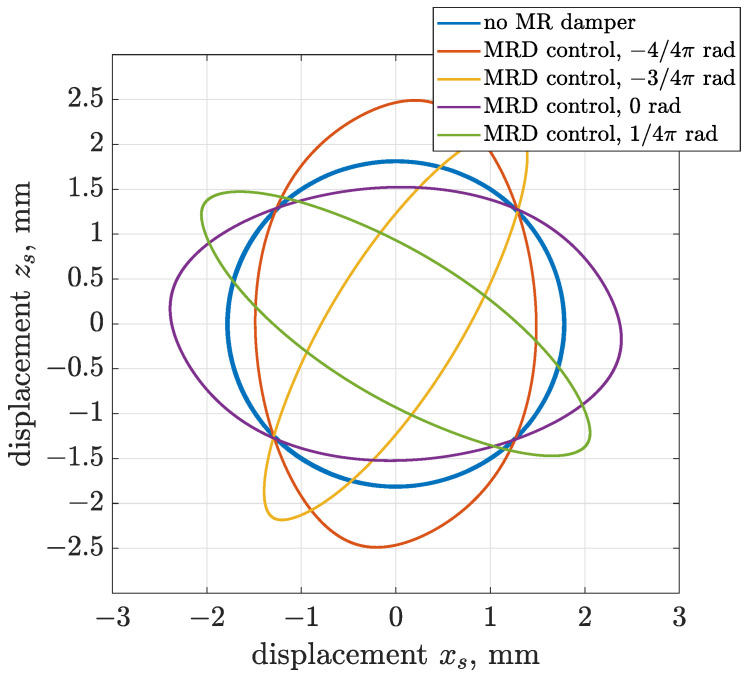
Different configurations of phase shift $\Delta \alpha_{alg}$ dedicated to semi-active generation of linear trajectories of vibration displacement using the dissipative domain of the Bouc–Wen MR damper model.

The trajectory control dedicated to MR damper was successively tested and presented in Figure 24 and Figure 25 for vibration acceleration and displacement, respectively. It can be indicated that the more applicable case of semi-active trajectory control, comparing to previous ideal active control, allows for modification of screen vibration trajectory from circular to linear. The obtained shapes of trajectories include a component of elliptical trajectory. It can also be stated that efficiency in the modification of trajectory depends on the desired algorithm phase shift. For phase shifts equal to 1/4 π or 5/4 π, the shape of the obtained trajectory is closer to linear in comparison to results obtained for phase shifts equal to 0 or π.

**Figure 26:**
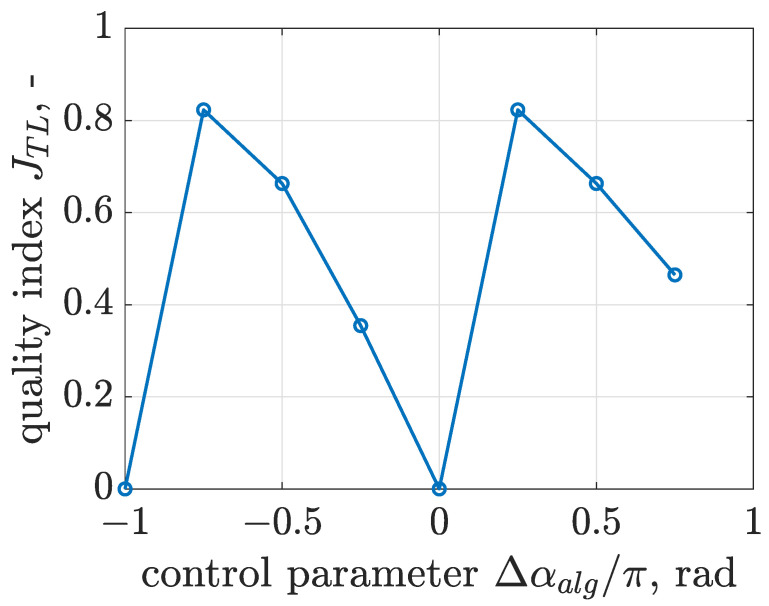
Comparison of values of quality index $J_{TL}$ assessing the linearity the trajectory control algorithm presented for different control parameters $\Delta \alpha_{alg}$ applied to a semi-active screen suspension with the Bouc–Wen model.

The quality of the proposed trajectory control algorithm was assessed for different values of control parameter $\Delta \alpha_{alg}$ in more a comprehensive approach based on a quality index of trajectory linearity denoted as $J_{TL}$, as presented in Figure 26. Its definition is mainly based on phase shift $\Delta \beta_{x,z}$ estimated between horizontal $x_{s}$ and vertical $z_{s}$ displacements using a cross-correlation function. Here, we recall for the reader that a zero phase shift results in a linear trajectory and a phase shift equal to π/2 corresponds to a circular trajectory, as discussed in Section 3. As as result, the quality index $J_{TL}$ was defined by the following:

(14) $$J_{TL}=\left|\cos\left(\Delta \beta_{x,z}\right)\right|\;\;\;\;\mathrm{where}\;\;\;\;\Delta \beta_{x,z}⟵\max\left(R_{x,z}\right),$$

where cross-correlation function of signals $x_{s}$ and $z_{s}$ is denoted as $R_{x,z}$.

The proposed quality index $J_{TL}$ can vary from 0 to 1; the greater the value of $J_{TL}$, the closer the generated vibration trajectory will be to the linear shape. Evaluated values of $J_{TL}$ indicate two optimized cases of linear trajectory within the analyzed range corresponding to $\Delta \alpha_{alg}$ equal to −3/4 π and 1/4 π rad, where $J_{TL}$ is close to 0.8. It can be noticed that the dependence of $J_{TL}$ on $\Delta \alpha_{alg}$ is a smooth function, where the worst case corresponds to $\Delta \alpha_{alg}$ equal to −π and 0 rad.

**Figure 27:**
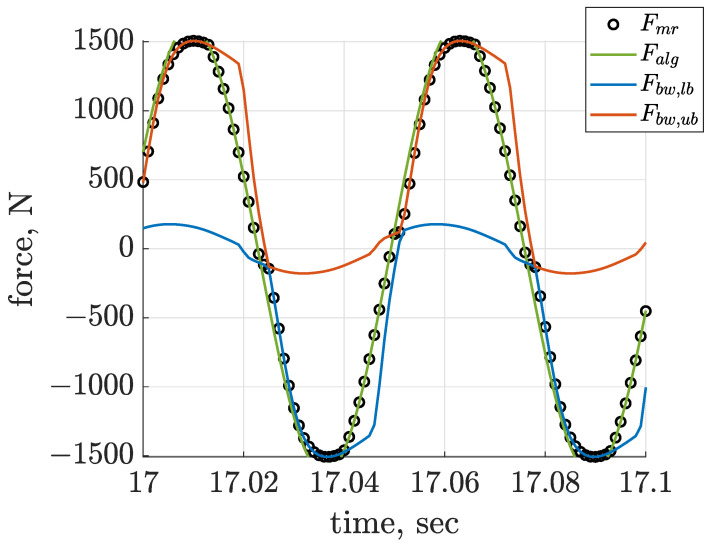
Quality of tracking of the horizontal desired control force $F_{alg,x}$ by the actual MR damper Bouc–Wen $F_{mr,x}$, accompanied by the lower $F_{bw,lb}$ and upper $F_{bw,ub}$ bounds, presented for the front screen suspension part for the control configuration of $\Delta \alpha_{alg}$ = −3/4 π rad.

**Figure 28:**
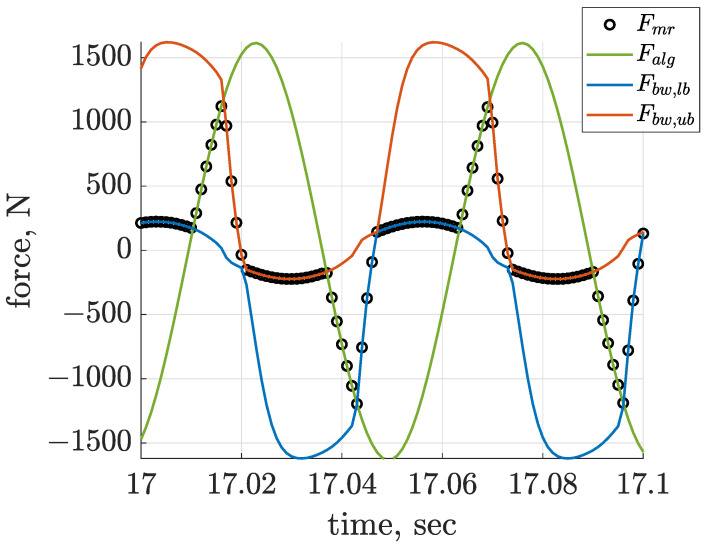
Quality of tracking of the vertical desired control force $F_{alg,z}$ by the actual MR damper Bouc–Wen $F_{mr,z}$, accompanied by the lower $F_{bw,lb}$ and upper $F_{bw,ub}$ bounds, presented for the front screen suspension part for the control configuration of $\Delta \alpha_{alg}$ = −3/4 π rad.

Further analysis gives insight into the results of linear trajectory generation based on a comparison of the desired control force $F_{mr}$ and the generated MR damper force $F_{alg}$. These signals are presented in Figure 27 and Figure 28 for the case of $\Delta \alpha_{alg}$ = −3/4 π rad with respect to the front suspension part, and they are accompanied with the corresponding limiting forces $F_{bw,lb}$ and $F_{bw,ub}$. The first Figure 27 shows the desired horizontal control force, which was almost ideally represented and generated by the MR damper model. Contrary to that, tracking of the desired vertical force by the actual MR damper force, which is presented in Figure 28, could only be achieved to a limited extent. Despite this fact, the high quality of linear trajectory generation according to the quality index $J_{TL}$ and displacement trajectories presented in Figure 25 was obtained for this case of $\Delta \alpha_{alg}$ and the proposed semi-active control.

An extended analysis was carried out for different control parameters $\Delta \alpha_{alg}$ and for each horizontally or vertically oriented MR damper applied to the selected front or rear part of the screen model. The quality of tracking the desired control force $F_{alg}$ by the MR damper Bouc–Wen models $F_{mr}$ was assessed based on a normalized quality index of force tracking denoted as $J_{FT}$, and results are listed in Table 2. The quality index $J_{FT}$ was defined based on a complement to the relative mean squared error calculated between the desired control force $F_{alg}$ and the actual MR damper force $F_{mr}$ as follows:

(15) $$J_{FT}=1-\frac{MSE_{FT}}{MSE_{FT,max}}\;\;\;\;\mathrm{where}\;\;\;\;MSE_{FT}=\frac{\sum_{j}{\left[F_{mr}\left(j\right)-F_{alg}\left(j\right)\right]}^{2}}{\sum_{j}{\left[F_{alg}\left(j\right)\right]}^{2}},$$

The symbol $MSE_{FT,max}$ denotes a normalization value which corresponds to the worst-case quality (maximum value) of force tracking evaluated over all considered control configurations and MR damper models. For a single control parameter $\Delta \alpha_{alg}$ and a single MR damper model, the maximum value of $MSE_{FT}$ was found by shifting the desired force $F_{alg}$ in time with respect to lower $F_{bw,lb}$ and upper $F_{bw,ub}$ force bounds, which resulted in a set of estimated ${\hat{F}}_{mr}$ time diagrams. As a result, the greater value of $J_{FT}$, the better the quality of force tracking achieved.

Control configurations which are in bold in Table 2 correspond to the best results with respect to trajectory linearity. It is indicated that the quality of force tracking for both the front and rear suspension parts was high for the selected MR damper model, whereas the second MR damper model oriented perpendicularly produced much greater tracking error. Furthermore, when control parameter $\Delta \alpha_{alg}$ was equal to −3/4 π or 1/4 π rad, the quality of force tracking for the horizontal or vertical MR damper, respectively, was significantly heightened.

Values of $J_{FT}$ varied from 0.052 for the rear vertical MR damper model and $\Delta \alpha_{alg}$ = −2/4 π rad to 0.999 for the rear horizontal MR damper model, and $\Delta \alpha_{alg}$ = −3/4 π rad. It is worth noting that high overall quality of trajectory linearity depends on dominant force tracking achieved for a single horizontal or vertical MR damper rather than on an average tracking quality: The control configuration corresponding to $\Delta \alpha_{alg}$ = −π rad have relatively high $J_{FT}$ values which varied from 0.424 to 0.476. However, it did not provide the best final result according to the displacement trajectories and $J_{TL}$ quality index. 

## 5. Conclusions

The article reported a trajectory control algorithm applied to a vibrating screen equipped with semi-active suspension. The proposed solution allows one to change the shape of the vibrating screen trajectory from circular to linear and control vibration amplitude. The reported results were obtained in simulations performed on the model developed and parameterized based on experimental data from a semi-industrial vibrating screen. The demonstrated solution was applied to the vibrating screen model with only one exciter. The main novelty of the reported solution is that according to the proposed control algorithm, the desired forces generated by MR dampers emulate an additional virtual mechanical exciter of the vibrating screen. In turn, it interacts with the existing exciter, resulting in conversion of the trajectory from circular to linear.

From the future application standpoint, the proposed online trajectory modification may lead to a different vibrating screen design. The desired operating trajectory must be maintained even in the presence of sudden and frequent changes in the processed material throughput or its physical properties (e.g., particles’ size distribution, particle shape or moisture). To tackle that problem, the modern vibrating screen’s mass is usually much higher then the mass of the processed material on deck to counteract the material flow changes. Lighter constructions with online motion control will require smaller and in turn more economic engines for vibration excitation. Finally, controlled, fast changes in the screen suspension stiffness will allow faster transitions through the resonant frequencies during startup and stop procedures, and may also be used for sieve cleaning. Future research will be focused on the experimental validation of the proposed trajectory control algorithm using different types of vibrating screens. The extension of the presented screen model to its dynamics in space will be also studied.

## Figures and Tables

**Table 1:** Estimated parameters of the simulator of the vibrating screen.

Model of Vibrating Screen			
$l_{er}$ = 0.011 m	$h_{er}$ = 0.048 m	$m_{s}$ = 102.6 kg	$I_{s}$ = 14.78 kgm${}^{2}$
$l_{sf}$ = 0.34 m	$h_{sf}$ = 0.14 m	$l_{sr}$ = 0.35 m	$h_{sr}$ = 0.08 m
$k_{sx}$ = 44,966 Nm${}^{-1}$	$k_{sz}$ = 57,094 Nm${}^{-1}$		
$c_{x_{s}}$ = 800 Nsm${}^{-1}$	$c_{z_{s}}$ = 800 Nsm${}^{-1}$	$c_{\varphi_{s}}$ = 31 Nms rad${}^{-1}$	$f_{\varphi_{s}}$ = 1.4 Nm
Model of Electric Motor and Mechanical Exciter			
$m_{e}$ = 32.4 kg	$I_{er}$ = 0.14 kgm${}^{2}$	$r_{e}$ = 0.007 m	$P_{m}$ = 0.75 kW
$p_{e}$ = 1.25	${\dot{\alpha }}_{m0}/2\pi$ = 25 Hz	${\dot{\alpha }}_{mn}/2\pi$ = 23.3 Hz	$s_{n}$ = 0.067
$M_{n}$ = 5.12 Nm	$M_{k}$ = 13.31 Nm	$M_{a}$ = 12.29 Nm	$s_{k}$ = 0.333
${\dot{\alpha }}_{m,th}$ = 2π 10.57 rads${}^{-1}$	$f_{\alpha_{m}}$ = 2.24 Nm		
Properties of Measurement Signals			
$f_{s}$ = 1000 Hz	$l_{a}$ = 0.051 m	$h_{a}$ = 0.117 m	
Bouc–Wen Model of the MR Damper			
$l_{mrf}$ = 0.229 m	$h_{mrf}$ = 0.040 m	$l_{mrr}$ = −0.111 m	$h_{mrr}$ = 0.169 m
$i_{mr,lb}$ = 0 A	$\alpha_{bw,lb}$ = 211,310	$\beta_{bw,lb}$ = −167,290	$A_{bw,lb}$ = 44,024
$\gamma_{bw,lb}$ = 32.6	$c_{bw,lb}$ = 878.0	$n_{bw}$ = 2	
$i_{mr,ub}$ = 1.0 A	$\alpha_{bw,ub}$ = 5840	$\beta_{bw,ub}$ = −4620	$A_{bw,ub}$ = 1216
$\gamma_{bw,ub}$ = 1351.0	$c_{bw,ub}$ = 1341.5		

**Table 2:** Quality index $J_{FT}$ assessing the tracking of the desired control force $F_{alg}$ by the actual force generated by MR damper Bouc–Wen models $F_{mr}$. Control configurations in bold correspond to the best quality index of trajectory linearity $J_{TL}$.

	Location of MR Damper Model: $J_{\mathrm{FT}}$			
$\Delta \alpha_{\mathrm{alg}}$	**Front Hor.**	**Front Vert.**	**Rear Hor.**	**Rear Vert.**
−4/4 π rad	0.458	0.476	0.473	0.424
**−3/4 π** **rad**	**0.994**	**0.272**	**0.999**	**0.131**
−2/4 π rad	0.953	0.129	0.919	0.052
−1/4 π rad	0.743	0.182	0.697	0.152
0 rad	0.430	0.466	0.390	0.448
**1/4 π** **rad**	**0.168**	**0.998**	**0.137**	**0.972**
2/4 π rad	0.079	0.945	0.074	0.968
3/4 π rad	0.165	0.748	0.168	0.760

## Nomenclature

*g*	gravitational acceleration
*x*, $\dot{x}$, $\ddot{x}$	horizontal displacement, velocity and acceleration with respect to the stationary frame of reference
*z*, $\dot{z}$, $\ddot{z}$	vertical displacement, velocity and acceleration with respect to the stationary frame of reference
($x_{s}$, $z_{s}$)	horizontal and vertical displacements of riddle center of mass
$\varphi_{s}$	inclination angle of the screen riddle with respect to the stationary frame of reference
$m_{s}$, $I_{s}$	mass and moment of inertia of the vibrating screen riddle
$M_{m}$, $\alpha_{m}$	torque and instantaneous angle of the electric motor with respect to the stationary frame of reference
($x_{er}$, $z_{er}$), ($x_{em}$, $z_{em}$)	horizontal and vertical displacements of the rotating shaft of the mechanical exciter and the unbalanced mass
$M_{e}$, $\alpha_{e}$	torque and instantaneous angle of the mechanical exciter with respect to the riddle frame of reference
$m_{e}$, $I_{er}$, $r_{e}$	mass and moments of inertia evaluated with respect to the rotating axis, distance from the axis of rotation to the center of unbalanced mass
($l_{er}$, $h_{er}$)	location of the exciter rotation axis with respect to the riddle center of mass and its frame of reference
($x_{sf}$, $z_{sf}$), ($x_{sr}$, $z_{sr}$)	horizontal and vertical displacements of front and rear parts of the screen suspension

$k_{sx}$, $k_{sz}$	horizontal and vertical stiffness of the single part of the screen suspension
$c_{x_{s}}$, $c_{z_{s}}$, $c_{\varphi_{s}}$, $f_{\varphi_{s}}$	horizontal and vertical viscous damping and angular viscous damping and dry friction related to the screen suspension
($l_{sf}$, $h_{sf}$), ($l_{sr}$, $h_{sr}$)	location of the suspension attachment points with respect to the riddle center of mass and its frame of reference
($x_{a}$, $z_{a}$)	horizontal and vertical displacement of the averaged sensor
($l_{a}$, $h_{a}$)	location of the averaged sensor with respect to the riddle center of mass and its frame of reference
$F_{alg}$, $F_{mr}$, $v_{mr}$	force desired by the control algorithm and actual force generated by the MR damper model, axial piston velocity of the MR damper model
($l_{mrf}$, $h_{mrf}$), ($l_{mrr}$, $h_{mrr}$)	location of the MR damper models attachment points with respect to the riddle center of mass and its frame of reference
$F_{bw,lb}$, $F_{bw,ub}$	lower and upper bounds generated by the Bouc–Wen model and set on the desired control force
$\Delta \alpha_{alg}$	parameter of trajectory control algorithm corresponding to the phase shift of the emulated mechanical exciter with respect to the real exciter
$J_{TL}$	quality index assessing linearity of screen vibration trajectory
$J_{FT}$	quality index assessing tracking of $F_{alg}$ by the $F_{mr}$
